# Chromatin accessibility contributes to simultaneous mutations of cancer genes

**DOI:** 10.1038/srep35270

**Published:** 2016-10-20

**Authors:** Yi Shi, Xian-Bin Su, Kun-Yan He, Bing-Hao Wu, Bo-Yu Zhang, Ze-Guang Han

**Affiliations:** 1 Key Laboratory of Systems Biomedicine (Ministry of Education) and Collaborative Innovation Center of Systems Biomedicine, Shanghai Center for Systems Biomedicine, Shanghai Jiaotong University, Shanghai, China; 2 Shanghai-MOST Key Laboratory for Disease and Health Genomics, Chinese National Human Genome Center at Shanghai, Shanghai, China

## Abstract

Somatic mutations of many cancer genes tend to co-occur (termed co-mutations) in certain patterns during tumor initiation and progression. However, the genetic and epigenetic mechanisms that contribute to the co-mutations of these cancer genes have yet to be explored. Here, we systematically investigated the association between the somatic co-mutations of cancer genes and high-order chromatin conformation. Significantly, somatic point co-mutations in protein-coding genes were closely associated with high-order spatial chromatin folding. We propose that these regions be termed Spatial Co-mutation Hotspots (SCHs) and report their occurrence in different cancer types. The conserved mutational signatures and DNA sequences flanking these point co-mutations, as well as CTCF-binding sites, are also enriched within the SCH regions. The genetic alterations that are harboured in the same SCHs tend to disrupt cancer driver genes involved in multiple signalling pathways. The present work demonstrates that high-order spatial chromatin organisation may contribute to the somatic co-mutations of certain cancer genes during tumor development.

Chromatin functions as a high-order structure that consists of the inheritable genomic DNA and genetic and epigenetic regulators, including proteins and RNAs. Studies in recent years have shown that the high-order spatial conformation of chromatin plays an important role in many nuclear processes, including DNA replication, gene expression regulation, and epigenetic organisation^1^,^2^,^3^,^4^,^5^,^6^,^7^,^8^. Recently, genome-wide chromatin conformation capture technology has been developed and applied to assess the spatial organisation of chromatin and has assisted researchers in gaining unprecedented insights into three-dimensional (3D) genome structures and their relationships to nuclear functions^6^,^9^,^10^,^11^.

In cancer research, somatic genomic aberrations, including single-nucleotide variances (SNVs), chromosome arrangements and translocations, and copy number alterations (CNAs), are well-known critical genetic events that are associated with tumor initiation and progression^12^. With regard to the relationship between genomic aberrations and chromatin structure, the accumulated data regarding structural variations in cancer genomes and the emergence of capture technology for assessing genome-wide chromatin conformation, including high-order chromatin conformation interaction (Hi-C) mapping, have allowed researchers to investigate these somatic genomic alterations with respect to genome-wide 3D chromatin conformation. Previous studies have indicated that chromosomal rearrangements are highly associated with spatial proximity^13^,^14^,^15^,^16^,^17^. Recently, the genome-wide association study of somatic translocation and Hi-C maps demonstrated the evidences supporting the “contact first” hypothesis^17^,^18^,^19^,^20^,^21^,^22^,^23^, that is, the paired genes of chromosomal translocation patterns co-localize in the nuclei of normal cell, prior to rearrangement^24^. For somatic CNAs and chromatin 3D association study, Fudenberg *et al.* suggested that the distribution of chromosomal alterations in cancer is spatially related to genomic architecture and can influence somatic CNAs during the evolution of cancer cells^25^.

The association between high-order chromatin conformation, somatic CNAs and chromosomal translocation has previously been proposed. However, whether spatial chromatin structure is also involved in somatic SNVs remains completely unclear. In a large majority of diagnosed cancer samples (patients), multiple somatic point mutations exist simultaneously and are herein called co-mutations. Many of these co-mutation events occur in a non-random fashion, and their occurrence can provide important information on the functional cooperation between mutated genes and their causal roles in carcinogenesis^26^. In cancer cells, some genes tend to be co-mutated, and others are rarely co-mutated. For example, in lung adenocarcinoma, compound *EGFR* mutations are frequently detected with co-mutations of other actionable genes, and these aberrations are associated with poor clinical outcomes^27^. Complex molecular genetic abnormalities involving three or more somatic mutations have also been reported in acute myeloid leukaemia^28^, upper tract urothelial carcinoma^29^, sun-exposed melanomas^30^, pulmonary mucinous adenocarcinoma^31^, and rectal cancer^32^.

The occurrence of somatic co-mutations of many cancer genes is widespread in tumourigenesis, and the mechanisms underlying these genetic events have yet to be explored. In this work, we collected somatic gene mutations from different cancer types from The Cancer Genome Atlas (TCGA)^33^, the Catalogue of Somatic Mutations in Cancer (COSMIC)^34^, and an available single cell sequencing data from prostate cancer^35^, and then compared the spatial proximity of the genes that are co-mutated with those that are not co-mutated. Here, we propose the hypothetical concept of Spatial Co-mutation Hotspots (SCHs), which represent spatially proximate chromatin loci that harbour genes that tend to be co-mutated during cancer initiation and progression. Additionally, we characterised SCHs derived from different cancer types, including their point mutation signatures, the conservation of flanking sequences of the point mutations, and the disruption of signalling pathways by driver mutations.

## Results

### Co-mutated gene pairs in cancers are spatially proximate in chromatin conformation

To survey the relationship between spatial chromatin structure and somatic SNVs in cancers, this study utilized data mining of Hi-C and somatic mutation data from cancer genomes. Several studies have previously revealed that the conformation of mammalian chromatin is conserved across cell types and, to some extent, even across species^1^,^6^,^8^. Therefore, we adopted Hi-C datasets from two human cell lines, diploid fibroblasts (IMR90) and embryonic stem cells (hESC)^1^, due to the lack of Hi-C data from cancer cells. For the somatic SNVs, we collected all SNVs from the TCGA and COSMIC databases and identified somatic point co-mutations contained in individual cancer samples. For a given cancer type, we calculated the 3D contact frequencies, which evaluate the spatial proximity of two genomic segments, of all of these paired co-mutated genes based on the Hi-C datasets. For each pair of mutated genes located on the same chromosome, we obtained their linear nucleotide distance. In this study, gene pairs that were located on different chromosomes were not considered due to the sparseness and low resolution of the inter-chromosomal Hi-C data. For a given linear nucleotide distance on a given chromosome, we calculated two types of backgrounds for spatial contact frequency: the overall background and the gene-level background. The overall background value *b*_*overall*_*(x)* for a given linear distance *x* on a given chromosome refers to the mean contact frequency of all of the paired chromatin fragments (bin size = 40k in this work) that are *x* nucleotides away from each other. The gene-level background value *b*_*gene*_*(x)* for a given linear distance *x* on a given chromosome refers to the mean contact frequency of all of the gene pairs whose transcription start sites are *x* nucleotides away from each other. As an alternative to overall background, gene-level background was also included in this study to achieve a fairer comparison to the co-mutated gene pairs. Additionally, for each co-mutated gene pair, in addition to *b*_*overall*_*(x)* and *b*_*gene*_*(x)* background values, we also collected the contact frequencies of the pairs of co-mutated genes.

**Figure 1:**
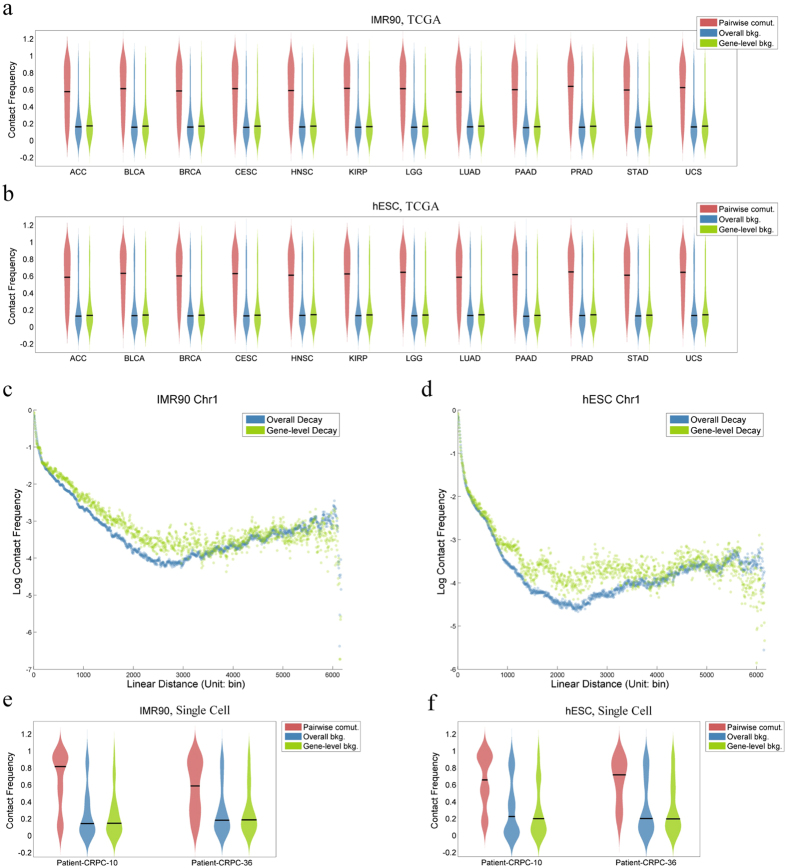
Spatial proximity of co-mutated gene pairs. Comparison of the contact frequency distribution between co-mutation pairs and overall background and gene-level background contact frequencies. All pairwise co-mutated contact frequency (Pairwise comut.) distributions were significantly higher than the overall background (Overall bkg.) values and the gene-level background (Gene-level bkg.) values, with all *P*-values < 10^−99^. (**a**) The three contact frequency distribution violin plots of the 12 TCGA cancers, based on IMR90 Hi-C data. (**b**) The three contact frequency distribution violin plots of the 12 TCGA cancers, based on hESC Hi-C data. (**c**) The overall background contact frequency decay function over the bin-level linear distance on IMR90 chromosome 1. (**d**) The overall background and gene-level background contact frequency decay scatter plots over the bin-level linear distance on hESC chromosome 1. Comparison of the contact frequency distribution between co-mutation pairs and overall background contact frequency and gene-level background contact frequency within single cells from two patients with prostate cancer based on IMR90 (**e**) and hESC Hi-C data (**f**).

We obtained three representative empirical distributions by concatenating the contact frequencies of all of the co-mutated gene pairs that occurred in 12 cancer types from TCGA and COSMIC based on IMR90 and hESC Hi-C data (Fig. 1a,b and Supplementary Figure 1a,b). As a control, both the overall background level and the gene-level contact frequency decay function over the bin-level linear distance on chromosomes were compared and were found to be similar, based on *IMR90* and *hESC* Hi-C data (Fig. 1c,d and Supplementary Figure 1c,d). Interestingly, the spatial proximities of co-mutated gene pairs in all 12 cancer types were significantly higher than the overall and gene-level backgrounds (paired *T*-test, all *P*-value < 10^−99^) (Fig. 1a,b and Supplementary Figure 1a).

Because the co-mutations of the paired genes that happen within the same sample may not necessarily occur in the same cell of the sample, therefore, to strengthen our hypothesis, we further analysed the spatial proximities of co-mutated gene pairs at single cell level. Based on availability, we collected a single cell somatic mutation dataset from a prostate cancer study^35^. As expectedly, the spatial proximities of the co-mutated gene pairs at single cell level were also significantly higher than the overall and gene-level backgrounds (Fig. 1e,f), indicating that the paired genes with co-mutations have spatial proximities in chromatin structure within the same cells.

**Figure 2:**
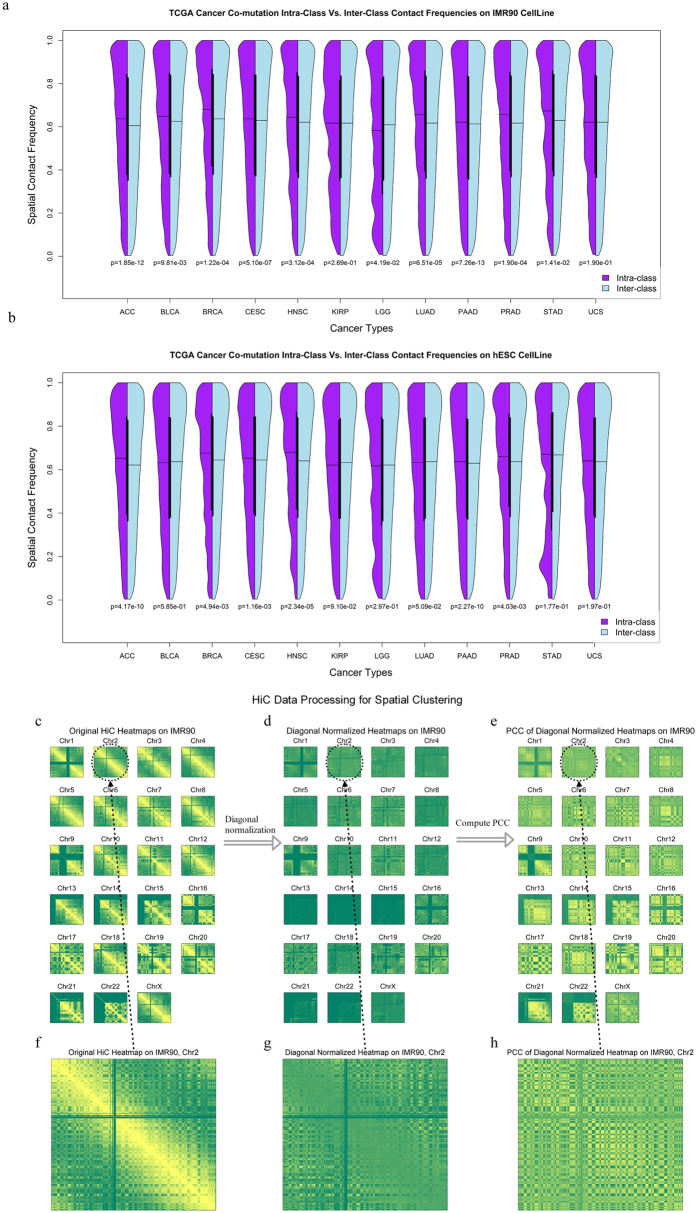
Spatial proximities of co-mutated gene clusters & Hi-C data processing. Comparison of intra- and inter-class contact frequency distributions. (**a**) The results of the 12 TCGA cancers, based on IMR90 Hi-C data. The left part (purple) of each violin plot is the intra-class contact frequency distribution. The right part (sky blue) of each violin plot is the inter-class contact frequency distribution (**b**) The results of the 12 TCGA cancers, based on hESC Hi-C data. (**c**) The original heatmaps of each chromosome were grouped based on the IMR90 Hi-C dataset and were then diagonally normalised for each chromosome (**d**). (**e**) The Pearson correlation coefficient (PCC) matrices were taken on the diagonal-normalised Hi-C heatmaps. (**f–h**) The zoomed-in matrices on chromosome 2 are shown as an example. We further overlapped these matrices with the co-mutated genes identified by TCGA/COSMIC datasets in sample matrices.

To determine whether somatic co-mutated gene pairs occurred in the same tumor samples, we further compiled genes by sample binary matrices for each cancer type from the TCGA and COSMIC databases. In such a matrix *A*, an element *a*_*ij*_= 1 indicates that the gene, *i*, is observed to be mutated in the sample, *j*, at least once, and *a*_*ij*_= 0 otherwise. Then, we clustered genes into different classes to identify the co-mutated gene pairs. The pairs of genes that were clustered into the same classes were called intra-class gene pairs and were considered to be more authentically co-mutated because their co-mutations were observed in multiple samples. The pairs of genes that were clustered into different clusters were called inter-class gene pairs and were considered as controls in the subsequent analyses. We then calculated all of the chromatin contact frequencies of the intra- and inter-class gene pairs and plotted their distributions side by side. Significantly, the *P*-value cut-off was set at 0.005, and the mean values of the contact frequencies of intra-class pairs were higher than those of the inter-class pairs in most cancer types, specifically including ACC, BLCA, BRCA, CESC, HNSC, LUAD, PAAD, PRAD, and STAD based on *IMR90* Hi-C data and ACC, BLCA, BRCA, CESC, HNSC, PAAD, and PRAD based on *hESC* Hi-C data (Fig. 2a,b for TCGA data and Supplementary Figure 2a,b for COSMIC data). These data suggest that in most cancer types, somatic co-mutated gene pairs have spatial proximities in chromatin structure.

### Identification of Spatial Co-mutation Hotspots (SCHs)

While we found that, on average, co-mutated genes are spatially proximate, a cluster of co-mutated genes may contain multiple sub-clusters that are located at different spatial chromatin loci on the given chromosomes. Thus, we propose the concept of SCHs, which are defined as spatial chromatin loci where certain genes that are spatially close to each other tend to be co-mutated during cancer initiation and progression. To obtain all SCHs, we overlapped the above gene clusters that were calculated based on the gene by sample matrices with the gene clusters that were calculated based on Hi-C data. When clustering genes based on the Hi-C data, we aimed to group genes that were scattered on a single chromosome in an unbiased fashion. Therefore, we normalised the original diagonal-dominated Hi-C map (Fig. 2c,f) and further processed it in a way that the chromatin contact frequencies were more uniformly distributed on the Hi-C map (Fig. 2d,e,g,h and Supplementary Figure 2c). We then clustered genes based on the processed Hi-C data to obtain spatial gene clusters and overlapped them with the above gene clusters obtained based on the co-mutated genes identified from the TCGA/COSMIC datasets via sample matrices. We considered the regions of spatial chromatin loci containing co-mutated gene pairs as SCHs. The number of SCHs differed among cancer types and Hi-C data and ranged from hundreds to thousands (Supplementary Table 1-1, 1-2, 1–3, 1–4). Each SCH may involve two to tens of genes.

### CTCF is enriched near SCH genes

**Figure 3:**
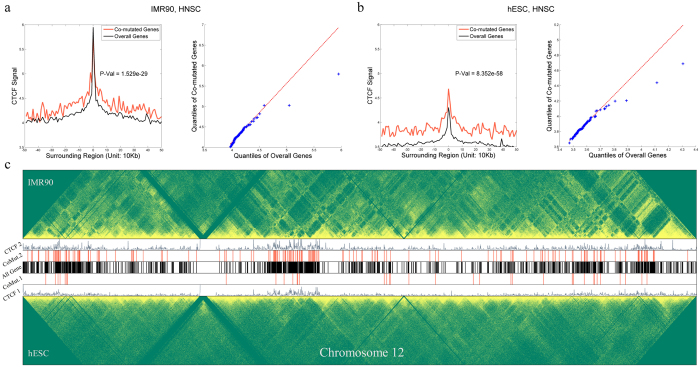
Co-mutated genes and CTCF. (**a**,**b**) Left: comparison of the CTCF ChIP-seq signals near the transcription start sites (TSSs) of the co-mutated genes in HNSC cancer (red) and on all genes for HNSC cancer (black), based on IMR90 and hESC Hi-C data, respectively. The T-test *P*-values are shown on the plots and indicate that CTCF enrichment was significantly stronger near co-mutated genes compared to genes in general; (**a**,**b**) Right: The corresponding quantile-quantile plot of the two CTCF distributions, which also demonstrates that the two CTCF distributions are different. (**c**) An example demonstrating the Hi-C heatmaps of the two cell lines and their corresponding CTCF signals, along with identified co-mutated genes and background genes. CTCF1 and CTCF2 represent the CTCF signals for the hESC and IMR90 cell lines, respectively. CoMut1 and CoMut2 represent the co-mutated genes for the two cell lines, respectively. The red and black bars indicate the TSS sites.

CTCF is known to play a critical role in chromatin high-order conformation^1^,^2^,^5^,^6^, e.g., in forming long-range chromatin loops or insulating epigenetic signals. Our previous research also showed that CTCF is enriched near chromatin topological domains^8^. In this study, we investigated whether CTCF signals were different between SCH genes and background (all genes). We found that for almost all of the 12 TCGA cancer types, the CTCF signals near the transcription start sites (TSSs) of SCH genes were significantly higher than those of the background, as shown in Fig. 3 and Supplementary Figure 3a,b. This observation led us to hypothesise that CFCF may be a key factor that contributes to gene co-mutations.

### Mutational signatures are similar within SCHs

**Figure 4:**
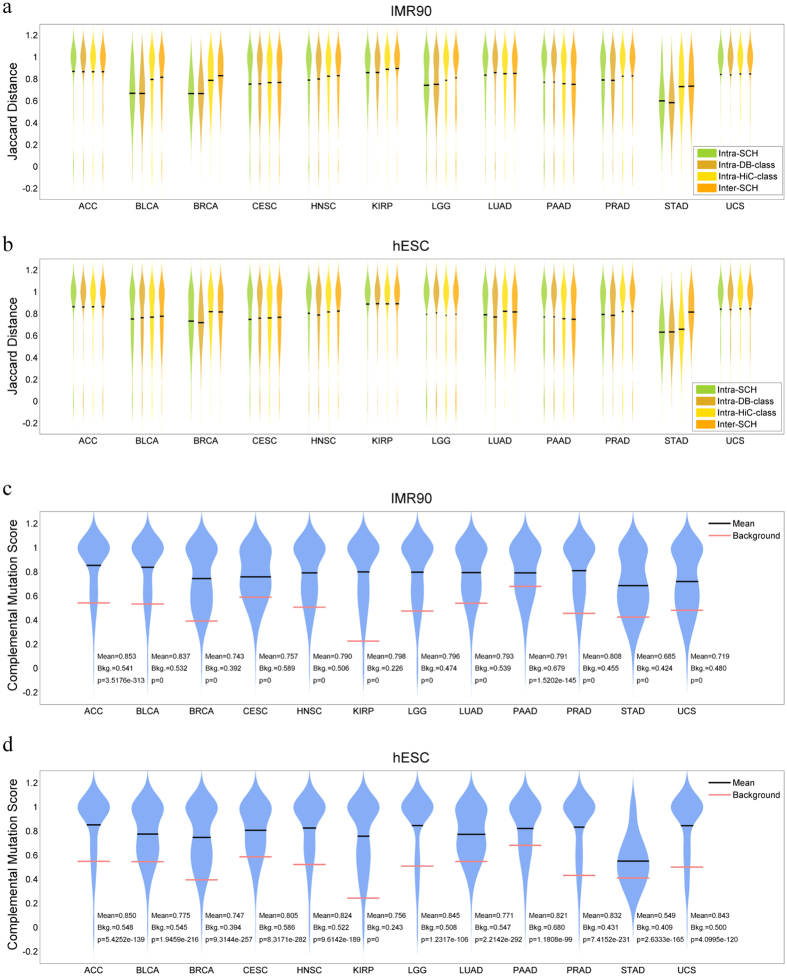
Mutational signatures in SCHs. The distributions of Jaccard distance of mutational signatures within SCHs (intra-SCH) were statistically compared with intra-DB-class, intra-Hi-C-class and inter-SCH for the 12 TCGA cancer types, based on IMR90 (**a**) and hESC Hi-C data (**b**), respectively. The lower the Jaccard distances, the more similar the mutation types. The score distributions of complementary mutational signatures of gene pairs within SCHs from 12 TCGA cancer types were statistically compared to that of all mutated genes, which served as the background score (Bkg). The analysis was based on IMR90 (**c**) and hESC Hi-C data (**d**), respectively. The *P*-values are indicated for the corresponding cancer types. The lower the complementary mutation scores, the more complementary the mutations to are each other.

To characterise the features of the mutational signatures of paired co-mutations genes with SCHs, we investigated the three types of gene datasets obtained from the above studies. Specifically, these datasets included the SCHs, the co-mutation gene pairs observed in multiple samples from the same cancer types contained in the TCGA and COSMIC database, herein called the database (DB) dataset, and the Hi-C gene dataset localised within the same chromatin loci based on Hi-C data from two human cell lines. In this study, we compared the mutational signatures of both intra- and inter-datasets. For a given gene dataset, we compiled a gene by mutation type matrix, *M*, with each element, *m*_*ij*_, indicating whether there was a type *j* mutation in gene *i*. Because there are 12 types of point mutations, each gene was represented as a vector with length equal to 12. We then computed and statistically analysed the Jaccard distances to measure the dissimilarity among these point mutation types in gene pairs within the SCH, DB and Hi-C datasets and between the SCH datasets (Fig. 4a,b, Supplementary Figure 4a,b and Supplementary Table 2). As shown in Fig. 4a,b, the Jaccard distance distributions indicated that, for most cancer types, the dissimilarities of mutation types of the intra-SCH cluster were significantly smaller than those of the intra-Hi-C and inter-SCH datasets and were also slightly smaller than those of the intra-DB cluster (*P*-values are shown in Supplementary Table 2). This finding suggests that the somatic point mutations within a given SCHs tend to be similar.

Next, we investigated whether the mutation types within the SCHs were, to a certain extent, complementary base pairs. Two mutations are complementary if their mutation directions are opposite, e.g., *A* > *T* and *T* > *A* transversions. For each SCH, we calculated a complementing score and also calculated the background complementing score over all of the SCH datasets. The detailed calculation methods are described in the Materials and Methods. Interestingly, the mutation types within a given SCH tended to be non-complementary, compared to the background, as determined by the *Z*-Test results (Fig. 4c,d and Supplementary Figure 4a,b). These data further support the observation that point co-mutation types in gene pairs within the given SCHs tend to be similar.

### Neighbouring sequences flanking co-mutation points within SCHs are conserved

**Figure 5:**
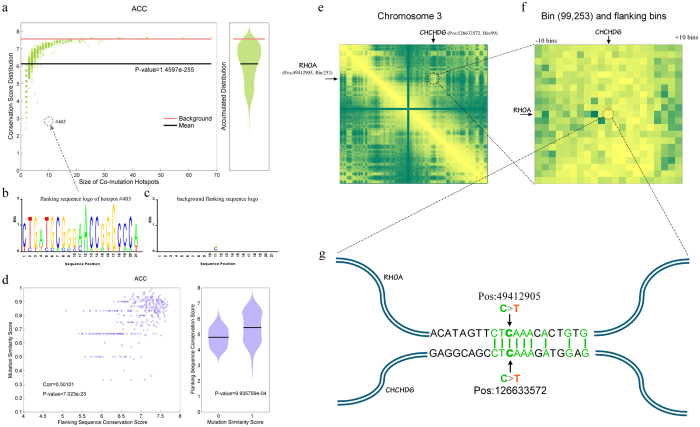
The conserved neighbouring sequences flanking the co-mutation points within SCHs. A typical example depicting the conservation of the neighbouring sequence flanking co-mutation points within SCHs. This example was derived from adrenocortical carcinoma (ACC) and was based on IMR90 Hi-C data. (**a**) The relationship (left) between the SCH sizes (x-axis) and the mean conservation scores of these flanking sequences (y-axis), where each green dot represents the mean conservation score of flanking sequences within a given SCH, the black line indicates the overall mean value and the red line represents the mean value based on the background. The lower the scores, the more highly conserved the flanking sequences. The violin plot of the accumulated conservation scores marginalised over SCH sizes (right). (**b**) The logo of the most conserved flanking sequences of SCH #405 is shown as an example. The mutation position corresponds to the 11th nucleotide. (**c**) The logo of the background flanking sequences was used as the control. (**d**) The correlation between the mutational signature similarities and the flanking sequence conservation within SCHs of ACC based on IMR90 Hi-C data (left). The comparison between the conservation of the flanking sequence and mutation similarity within SCHs was scored as 0 and 1, where 0 means identical and 1 means distinct. (**e–g**) The mutational signatures and flanking sequences of both RHOA and CHCD6 within the corresponding bin of chromosome 3 showed conservation. The original heatmap (**e**) and the magnified 21-by-21 bin of the heatmap (**f**) showing the co-mutation of RHOA and CHCD6 within the same SCH. Their mutational signatures, positions and flanking sequences (**g**) are also shown.

Because the point co-mutation types in a SCH tend to be similar, we next sought to determine whether the genomic DNA sequences flanking the mutation points are also conserved to some extent. Therefore, we collected all of the genomic DNA sequences that flanked the mutation points by 10 nucleotides in both directions, such that each sequence contained 21 nucleotides, and the mutation point was indexed as 11. For groups of such sequences belonging to the same SCHs, we aligned them and computed a sequence conservation score (Supplementary Table 3). We then statistically compared the distribution of these scores with the background conservation score. Interestingly, the sequences flanking mutation points in the same SCHs were significantly conserved compared to the background score, as determined by *Z*-Test (Fig. 5a,b and Supplementary Figure 5a,b).

We further investigated the correlation between the similarity of point co-mutation types within SCHs and the conservation of their flanking sequences. More interestingly, we found that, in most cancer types, if the mutation type within an SCH was similar, their flanking sequences tended to be significantly more conserved (Fig. 5d and Supplementary Figure 5c,d). For example, Fig. 5e shows the original Hi-C heatmap of chromosome 3 with two point mutations on *RHOA* and *CHCD6*, with their corresponding bins indicated based on *IMR90* data. Figure 5f shows a higher magnification of the 21-by-21 bin heatmap surrounding the bin pair containing *RHOA* and *CHCHD6*. Figure 5g demonstrates the positions of the two mutations on the spatially proximate gene pair <*RHOA* and *CHCHD6*> and their flanking sequences, indicating that they have the same mutation type and similar flanking sequences. This phenomenon could be shaped by some protein complexes, such as replication-associated proteins, transcription factors, and the insulator protein, CTCF, under certain spatial microenvironment conditions that affect the replication machinery within different SCHs, which could lead to different types of point co-mutations during tumorigenesis.

### Co-mutations of gene pairs within SCHs may disrupt distinct molecular signalling pathways

**Figure 6:**
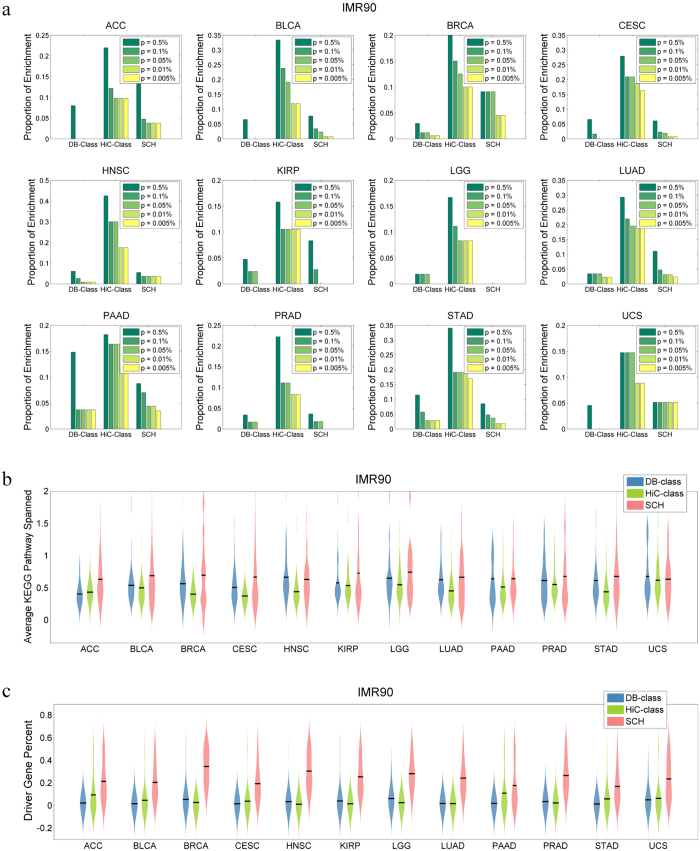
Pathway enrichment and cancer driver genes within SCHs. (**a**) The proportions of KEGG signalling pathway enrichment among different gene clusters from the 12 TCGA databases (DB), IMR90 Hi-C data and SCHs (overlap) were statistically compared, with different significance cut-offs. The average KEGG pathways represented (**b**) and the percentages of cancer driver genes (**c**) among the three datasets were also statistically compared for the 12 TCGA cancer types.

We assumed that under normal physiological conditions, the co-mutation rate is very low within chromatic loci. However, under certain conditions, the occurrence of co-mutations of gene pairs with SCHs could be evolutionally selected, especially if the cooperation of these co-mutation events may confer cancer cells with advantages in survival, growth and metastasis. To confirm this hypothesis, we sought to determine the extent to which biological pathways are enriched in the three types of gene datasets. Based on the *KEGG Homo Sapiens* pathway database^36^, we computed a hypergeometric score to statistically evaluate the enrichment of *KEGG* signalling pathways for each gene dataset^37^. By setting different *P-value* cut-offs, we computed the percentages of enrichment of these co-mutated genes (Fig. 6a and Supplementary Figure 6). The genes from the DB dataset were less likely to be enriched in *KEGG* signalling pathways, while the genes that clustered in the Hi-C dataset, which are spatially proximate but not necessarily co-mutated, were most likely to be enriched in *KEGG* pathways. Interestingly, for the genes within SCHs, which were the subsets with co-mutated gene pairs derived from the DB dataset, the percentages of *KEGG* pathway enrichment were significantly increased, compared to that of genes in the DB dataset (Fig. 6a and Supplementary Figure 6a). Supplementary Table 5-1 lists all the pathways enriched on each co-mutation hotspot as well as some basic statistics; the items are sorted according to the hypergeometric significant *P*-values. Supplementary Table 5-2 lists the pathways sorted according to their occurrence in multiple cancers, as well as detailed occurrences in SCH for each cancer and HiC cell lines. From Supplementary Table 5-2, we found that many high occurrence pathways are indeed related to the famous Pan-Cancer hallmarks^38^,^39^. For example, the pathway ECM_RECEPTOR_INTERACTION appears in 9 out of 12 cancer types; it is highly related to cell migration, differentiation, proliferation, and apoptosis, which correspond to the “cell immortality” and “metastasis” cancer hallmarks^38^. The pathway SYSTEMIC_LUPUS_ERYTHEMATOSUS appears in 8 out of 12 cancer types, which is related to the cancer hallmark “evading the immune system”^38^. The pathway PURINE_METABOLISM appears in half of the cancer types which is related to the cancer hallmark “abnormal metabolic pathways”^38^.

We further analysed the distributions of the numbers of *KEGG* pathways spanned by the three types of datasets. Significantly, the genes within SCHs spanned more *KEGG* signalling pathways in most cancer types, although the size of the SCH dataset was smaller than the DB or Hi-C datasets, as it represented the overlap between the latter two (Fig. 6b and Supplementary Figure 6b). This observation of SCH KEGG coverage suggests that co-mutations of genes within SCHs may alter many *KEGG* pathways that synergistically promote tumorigenesis.

We also evaluated the distributions of candidate cancer driver genes based on the Cancer Gene Census deposited in COSMIC database in the three types of datasets. Surprisingly, in all of the 12 TCGA cancer types, the percentages of driver genes in SCHs were significantly higher than those in both DB and Hi-C data (Fig. 6c and Figure S6c, Supplementary Table 4-1, 4-2) This difference suggests that spatially proximate co-mutated genes within the same chromatin loci may tend to be driver genes, whose mutations have been causally implicated in cancer.

## Discussion

In this work, we systematically investigated the associations between somatic point co-mutations of protein-coding genes in different cancer types and high-order genome conformation. We found that these co-mutated genes are generally spatially proximate and tend to be distributed on the same chromatin loci; therefore, we propose to term these regions SCHs. These SCHs share some common features, including similar mutational signatures, conserved neighbouring sequences flanking the co-mutation points, and capable of disrupting genes involved in distinct molecular pathways.

The reason why SCHs occur in cancer should be further investigated. The SCHs are a subset of the Hi-C interaction maps, which were based on two Hi-C datasets obtained from *IMR90* or *hESC* cell lines. In addition to spatial proximity in chromatin structure, the same SCHs have similar mutational signatures and conserved neighbouring sequences flanking these co-mutation points, and the degree of conservation is positively correlated with the similarity in mutational signatures. For 11 of the 12 TCGA cancer types, with the exception of stomach adenocarcinoma, the similarity of the mutational signatures and the degree of conservation of the flanking DNA sequences were highly correlated (Fig. 5d and Supplementary Figure 5c,d). This correlation suggests that the non-randomness of co-mutations of gene pairs within SCHs may be associated with their flanking sequences with respect to spatial genome conformation. Thus, we speculate that an unknown mechanism leads to simultaneous or successive point co-mutations of gene pairs due to chromatin topological accessibility and homologous DNA sequences within SCHs.

Different cancer types showed different SCH sets, and the numbers of SCHs and genes contained in a given SCH were also very different between cancer types (Supplementary Table 1-1). These differences could have been due to the three following reasons: (1) the different availability of data from different cancer types; (2) the local differences in chromatin conformation for a given specific cancer cell, although in general, the chromatin conformation for both *IMR90* and *hESC* cell lines was conserved; and (3) in different cancer types, different aetiologies and carcinogenic mechanisms, such as small compounds, virus infection, and genetic defects, could affect different spatial chromatin loci.

These co-mutations in SCHs may exert synergistic effects on cancer initiation and progression, possibly by altering distinct molecular signalling pathways. Here, we showed that SCHs tend to span more *KEGG* signalling pathways and disrupt more cancer driver genes. If the co-mutated genes are calculated only based on gene by sample matrixes (DB datasets) from the TCGA or COSMIC databases, without considering Hi-C data, the genes obtained do not tend to be enriched in *KEGG* pathways. However, if these genes are spatially close to each other, as determined from the Hi-C data, the probability that they are involved in *KEGG* pathways is significantly increased (Fig. 6a and Supplementary Figure 6). Specifically, the spatially proximate co-mutated genes within SCHs that overlapped between the DB and Hi-C datasets tended to span significantly more *KEGG* signalling pathways (Fig. 6b and Supplementary Figure 6). If these genes were both co-mutated and spatially proximate, their chance of being driver genes was significantly increased (Fig. 6c and Supplementary Figure 6). Taken together, we conjecture that, if cancer genes with co-mutations are spatially proximate, they may exert synergistic roles in tumorigenesis, as they may cause multiple hits on cooperative biological processes and signalling pathways, thereby conferring cancer cells with advantages, such as growth and evolution.

However, it should be noted that, in this study, we defined co-mutations as more than two mutations of genes occurring in the same cancer sample, which is not very rigorous. A more rigid definition of co-mutations would be mutations that occur in the same cells of a given patient sample. With this definition in mind, it would be interesting to combine single-cell DNA sequencing and Hi-C technologies, which would allow us to rigorously define SCHs corresponding to different cancer sub-clones and subsequently to explore cancer clonal evaluation under the perspective of high-order single-cell chromatin conformation.

## Methods

### Somatic mutation data and DB clustering

For the somatic mutation data used in this work, we downloaded all “IlluminaGA DNASeq Curated” somatic mutations from TCGA, which covered 12 cancer types, and all somatic mutations from COSMIC, which covered 43 cancers. While all of the datasets of the 12 TCGA cancer types were used in this study, due to variations in dataset quality and quantity, only 30 datasets from COSMIC cancer types were used. Considering that the curated TCGA data contain less noise, we only presented results obtained based on the TCGA datasets in the main text, while the COSMIC-based results are shown in the Supplementary materials. Both the TCGA and COSMIC datasets that we download were timestamped April 8^th^, 2015; the datasets have not been updated since then. The somatic point mutation data from single cells are obtained from circulating tumor cells of patients with prostate cancer^35^. Here we adopted the whole-exome sequencing data of two patients CRPC-10 and CRPC-36, with total number of 111 and 248 somatic mutations, respectively^35^.

For the obtained somatic mutation data from the TCGA and COSMIC datasets, we first re-organised the data into gene by sample binary matrices. In such a matrix *A*, an element *a*_*ij*_ = 1 indicates that the gene, *i*, is observed to be mutated in the sample, *j*, at least once, and *a*_*ij*_ = 0 otherwise. We then ranked the genes in descending order, according to the number of samples in which the genes were observed to be mutated. Then, similar to an previously proposed clustering approach^40^, we iteratively took the top-ranked gene that had yet to be clustered as the seed gene (the seed of a new cluster) and included the following un-clustered genes as long as their normalised Hamming similarity values^41^,^42^, defined as


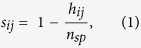


were greater than or equal to a given threshold. In this equation, *s*_*ij*_ denotes the Hamming similarity between the *i*th and the *j*th genes, while *h*_*ij*_ is the Hamming distance^43^ between this gene pair and *n*_*sp*_is the overall number of samples. For the best threshold, we tested 0.1, 0.2, …, 1.0 and chose the best threshold if the corresponding 3D contact frequencies between the intra- and inter-clusters were most significant according to the unpaired two-sample *T*-test, which is a standard significant test if the two testing populations have different sample size^44^. Note that we did not necessarily prefer the greater intra-cluster contact frequencies than the inter-cluster contact frequencies, and we only chose the best threshold according to the significance value of the unpaired *T*-Test between these two vectors^44^.

### Hi-C data and Hi-C clustering

We downloaded the public Hi-C data of the two cell lines, *IMR90* (human fibroblast cells) and *hESC* (human embryo stem cells), which were generated by Bin Ren’s lab^1^. We organised the downloaded raw data into matrices, such that each element (bin) in such a matrix represents the contact frequency between two 40k-nucleotide-long chromatin segments. As such, for each cell line, we obtained 23 contact frequency matrices, corresponding to the 23 human chromosomes. Note that we did not consider inter-chromosomal contact frequencies due to the sparseness and the computational complexity issues caused by the scale of the data.

To calculate the overall background contact frequency of a given linear distance, *d*, we used the equation,


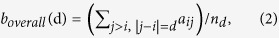


where *n*_*d*_ is the number of elements in the *d*th off-diagonal vector of the contact frequency matrix *A*. To calculate the gene-level background contact frequency of a given linear distance, *d*, we followed the same steps that were used for the overall background calculation but only included the elements in *A* that contained gene pairs.

When clustering genes for each chromosome based on the original Hi-C matrices, the genes that were linearly close tended to cluster together (diagonal dominant effect), while the genes that were linearly far apart, but were still significantly proximate compared to the corresponding background contact frequency, were very unlikely to cluster together. Another obstacle was that there were many missing values in the original Hi-C matrix. To alleviate the diagonal dominant effect and to circumvent the missing value problem, we used the two following steps: Step 1: We normalised the original Hi-C matrix *A* by dividing each contact frequency by its corresponding off-diagonal mean contact frequency. However, after this step, the contact frequencies close to the main diagonal were somehow over-penalised, and the missing values still existed. Here, we denoted the new matrix *A*’ with each element *a*_*ij*_’. Step 2: For each diagonal-normalised contact frequency, *a*_*ij*_’, we calculated a Pearson correlation coefficient, *c*_*ij*_, between the *i*th and *j*th column vectors of *A*’. After these two steps, the diagonal-dominant effect was minimised, and the missing value problem was addressed, except for cases in which the entire column was missing values (See Fig. 3 and Supplementary Figure 3). The correlation coefficient matrix *C* was then used for gene clustering. We use a hierarchical clustering algorithm, with cut-off equals to


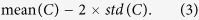


### CTCF ChIP-seq data

The CTCF data used in this work were downloaded from the ENCODE ChIP-seq database^45^. Specifically, the accession IDs for IMR90 and hESC CTCF data were “wgEncodeEH002831”^46^ and “wgEncodeEH000085”^45^, respectively.

### Similarity of mutational signatures

For a given cluster of genes, we first compiled a gene by 12 mutational type matrix *M*, with each element, *m*_*ij*_, indicating the number of type *j* mutations in the gene, *i*. A mutational type is one of *NT*_*j*_ > *NT*_*j*_, where *NT*_*i*_ ≠ *NT*_*j*_ ∈ {*A, T, C, G*}. We then replaced any mutation count greater or equal to 1 to obtain the matrix’s binary version, *M’*. Each gene was represented by a binary vector with a length of 12. Based on this vector representation, we calculated the Jaccard distances^48^ for all of the gene pairs and reported the mean Jaccard distance for each cluster. The smaller the Jaccard distance, the more similar the co-mutational signature of gene pairs within a cluster.

### Complementary score analysis

For a gene by mutational type matrix *M* corresponding to a gene cluster, *G*, we computed a complementing mutation score, which was defined as follows:


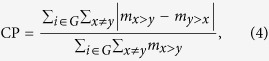


where *x, y* ∈ {*A, T, C, G*}. The background complementary score was calculated in the same way but was based on a combined whole cluster. The significance *P*-values was obtained based on the widely adopted *Z*-test.

### Pathway enrichment test

For a given cluster containing *n* genes, the probability of having *r* genes involved in the same functional categories in this cluster with a total of *N* genes was computed using the hypergeometric function as follows:


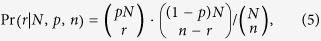


where *p* is the percentage of the genes assigned to functional categories, with respect to all genes reported in the *KEGG* pathway database^37^.

This probability was taken as the *P*-value of *KEGG* pathway enrichment of these assigned genes within a given cluster. The *P*-value of a cluster was defined as the smallest *P*-value over all pathways. The smaller the *P*-value of a cluster, the more likely that the genes would be assigned to the same *KEGG* pathway. For the three cluster types, we calculated the fraction of clusters whose *P*-values were smaller than a significant cut-off, i.e., the number of significant clusters divided by the total number of clusters.

## Additional Information

**How to cite this article**: Shi, Y. *et al.* Chromatin accessibility contributes to simultaneous mutations of cancer genes. *Sci. Rep.* **6**, 35270; doi: 10.1038/srep35270 (2016).

## Supplementary Material

Supplementary Information

Supplementary Table 1-1

Supplementary Table 1-2

Supplementary Table 1-3

Supplementary Table 1-4

Supplementary Table 2

Supplementary Table 3

Supplementary Table 4-1

Supplementary Table 4-2

Supplementary Table 5-1

Supplementary Table 5-2
