# Supplementary material for: Chromatin accessibility contributes to simultaneous mutations of cancer genes
Source: Sci Rep. 2016 Oct 20;6:35270. doi: 10.1038/srep35270 (PMC5071887; doi:10.1038/srep35270)
Supplement: Supplementary Information [file srep35270-s1.pdf]

Chromatin accessibility contributes to simultaneous mutations of cancer genes

Yi Shi[1,†], Xian-Bin Su[1,†], Kun-Yan He[1], Bing-Hao Wu[1,2], Bo-Yu Zhang[1], Ze-Guang Han[1,2*]

[1]Key Laboratory of Systems Biomedicine (Ministry of Education) and Collaborative Innovation Center of Systems Biomedicine, Shanghai Center for Systems Biomedicine, Shanghai Jiaotong University, Shanghai, China

[2]Shanghai-MOST Key Laboratory for Disease and Health Genomics, Chinese National Human Genome Center at Shanghai, Shanghai, China

[†]These authors contributed equally to this work.

*To whom correspondence should be addressed: Ze-Guang Han, Key Laboratory of Systems Biomedicine (Ministry of Education) and Collaborative Innovation Center of Systems Biomedicine, Shanghai Center for Systems Biomedicine, Shanghai Jiaotong University, Shanghai, China; 800 Dongchuan Road, Minhang District, Shanghai 200240, China. Tel: 86 (21) 34207304; Fax: 86 (21) 34206059; Email: hanzg@sjtu.edu.cn

Supplementary Figures

a

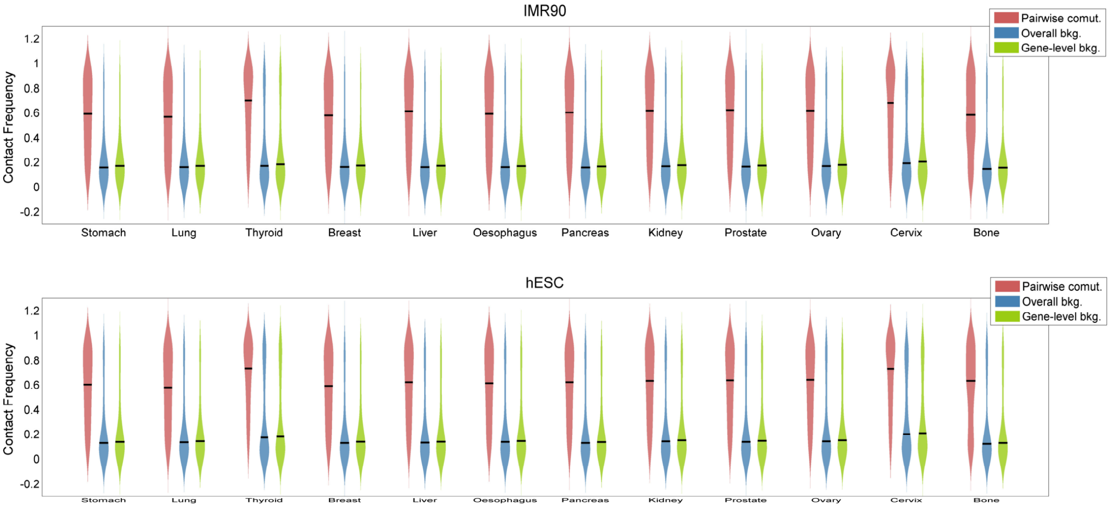

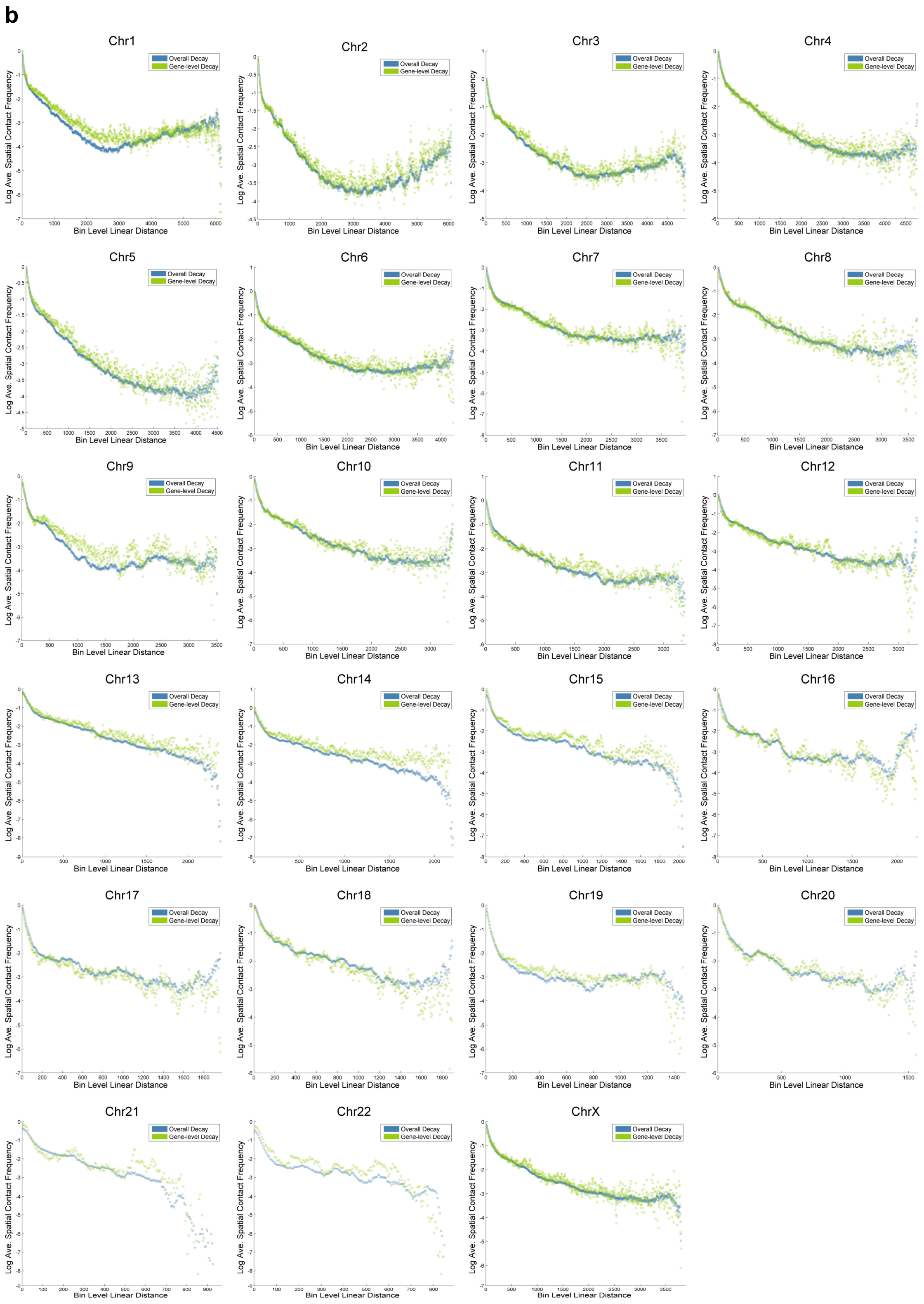
b
Chr1
Chr2
Chr3
Chr4
Chr5
Chr6
Chr7
Chr8
Chr9
Chr10
Chr11
Chr12
Chr13
Chr14
Chr15
Chr16
Chr17
Chr18
Chr19
Chr20
Chr21
Chr22
ChrX
Overall Decay
Gene-level Decay
Log Ave. Spatial Contact Frequency
Bin Level Linear Distance

c

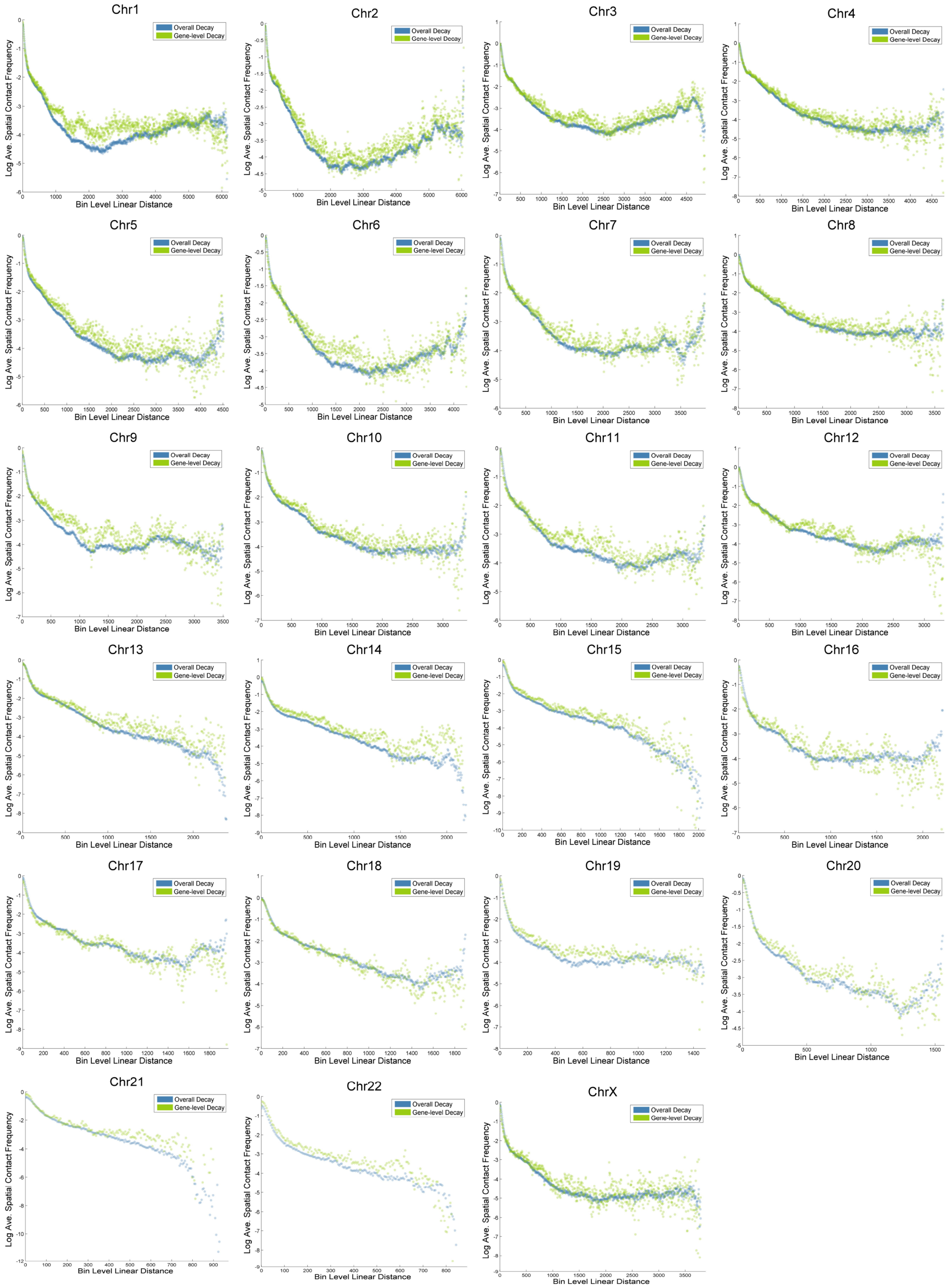

Supplementary Figure 1. Spatial proximity of co-mutated gene pairs. **(a)**. Comparison of the contact frequency distribution between co-mutation pairs and overall background contact frequency and gene-level background contact frequency in the two Hi-C cell lines and 12 TCGA cancer types. All pairwise co-mutated contact frequency (Pairwise comut.) distributions are significantly higher than the overall background (Overall bkg.) values and gene-level background (Gene-level bkg.) values, with all P-values < 10^{-99}. **(b)**. Overall

background and gene-level background contact frequency decay scatter plots over bin-level linear distance in the IMR90 cell line. **(c)**. Overall background and gene-level background contact frequency decay scatter plots over bin-level linear distance in the hESC cell line.

a

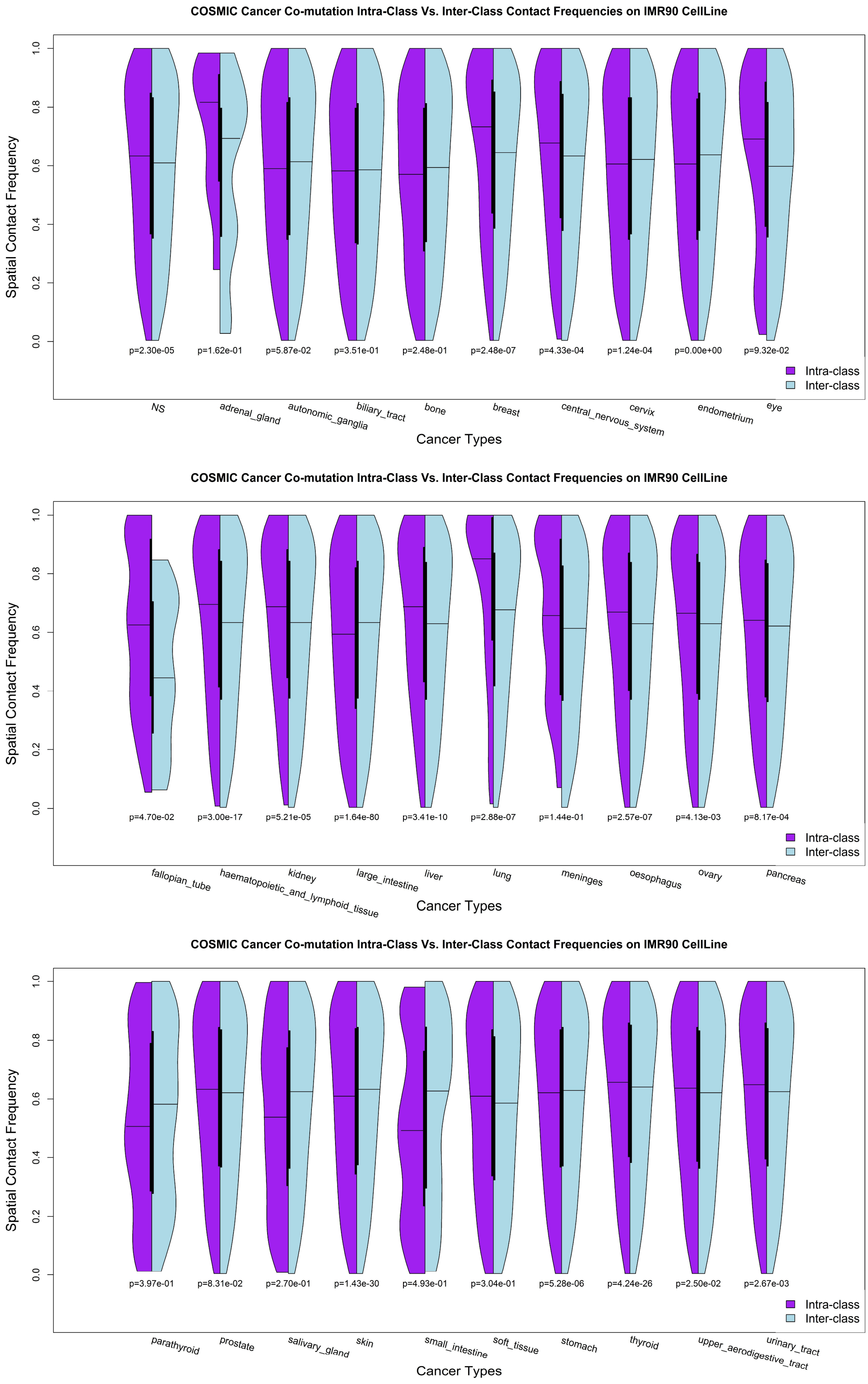

COSMIC Cancer Co-mutation Intra-Class Vs. Inter-Class Contact Frequencies on IMR90 CellLine
Spatial Contact Frequency
0.0
0.2
0.4
0.6
0.8
1.0
p=2.30e-05
p=1.62e-01
p=5.87e-02
p=3.51e-01
p=2.48e-01
p=2.48e-07
p=4.33e-04
p=1.24e-04
p=0.00e+00
p=9.32e-02
Intra-class
Inter-class
NS
adrenal_gland
autonomic_ganglia
biliary_tract
bone
breast
central_nervous_system
cervix
endometrium
eye
Cancer Types
COSMIC Cancer Co-mutation Intra-Class Vs. Inter-Class Contact Frequencies on IMR90 CellLine
Spatial Contact Frequency
p=4.70e-02
p=3.00e-17
p=5.21e-05
p=1.64e-80
p=3.41e-10
p=2.88e-07
p=1.44e-01
p=2.57e-07
p=4.13e-03
p=8.17e-04
Intra-class
Inter-class
fallopian_tube
haematopoietic_and_lymphoid_tissue
kidney
large_intestine
liver
lung
meninges
oesophagus
ovary
pancreas
Cancer Types
COSMIC Cancer Co-mutation Intra-Class Vs. Inter-Class Contact Frequencies on IMR90 CellLine
Spatial Contact Frequency
p=3.97e-01
p=8.31e-02
p=2.70e-01
p=1.43e-30
p=4.93e-01
p=3.04e-01
p=5.28e-06
p=4.24e-26
p=2.50e-02
p=2.67e-03
Intra-class
Inter-class
parathyroid
prostate
salivary_gland
skin
small_intestine
soft_tissue
stomach
thyroid
upper_aerodigestive_tract
urinary_tract
Cancer Types

b

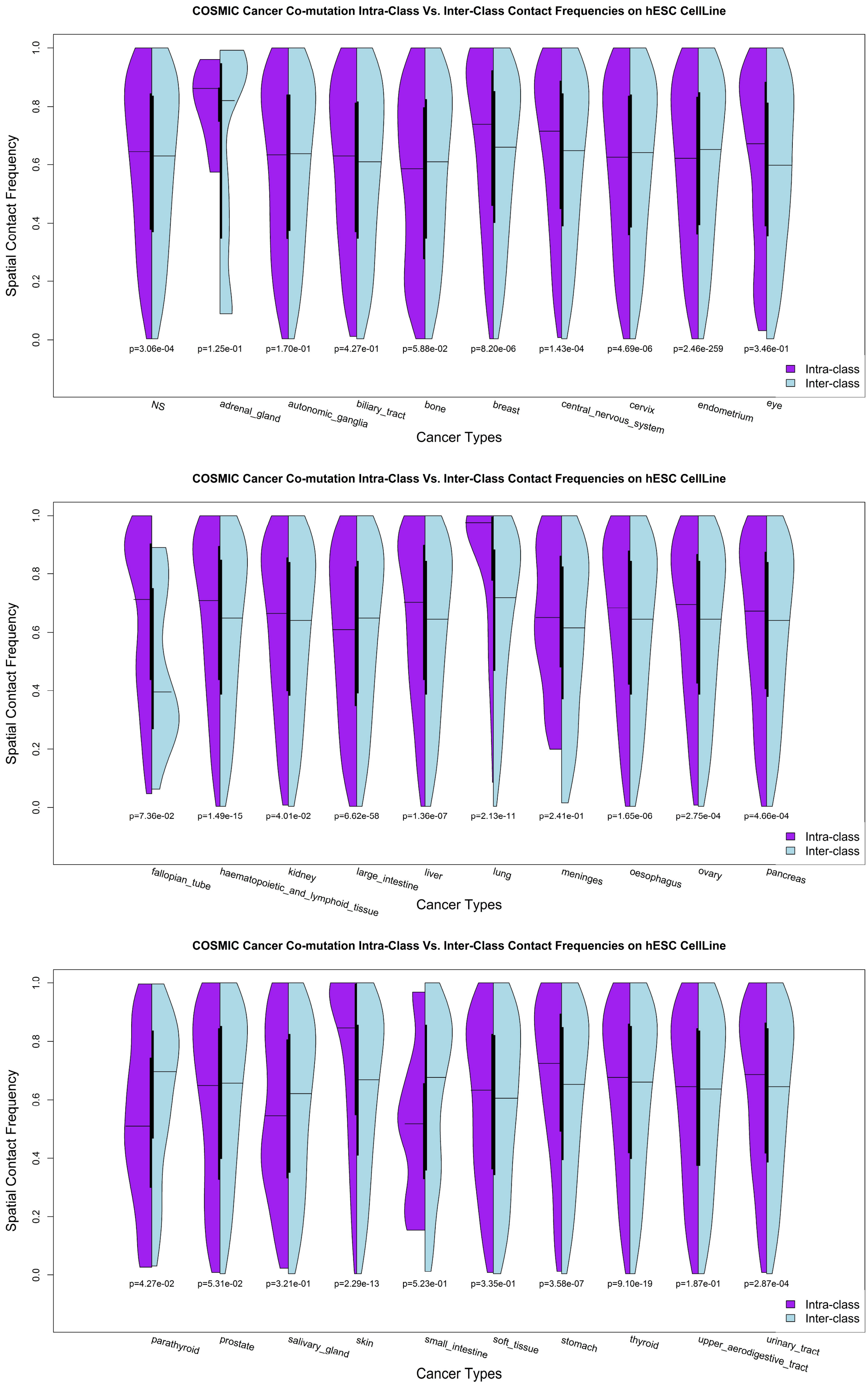
COSMIC Cancer Co-mutation Intra-Class Vs. Inter-Class Contact Frequencies on hESC CellLine
Spatial Contact Frequency
1.0
0.8
0.6
0.4
0.2
0.0
p=3.06e-04
p=1.25e-01
p=1.70e-01
p=4.27e-01
p=5.88e-02
p=8.20e-06
p=1.43e-04
p=4.69e-06
p=2.46e-259
p=3.46e-01
Intra-class
Inter-class
NS
adrenal_gland
autonomic_ganglia
biliary_tract
bone
breast
central_nervous_system
cervix
endometrium
eye
Cancer Types
COSMIC Cancer Co-mutation Intra-Class Vs. Inter-Class Contact Frequencies on hESC CellLine
Spatial Contact Frequency
p=7.36e-02
p=1.49e-15
p=4.01e-02
p=6.62e-58
p=1.36e-07
p=2.13e-11
p=2.41e-01
p=1.65e-06
p=2.75e-04
p=4.66e-04
Intra-class
Inter-class
fallopian_tube
haematopoietic_and_lymphoid_tissue
kidney
large_intestine
liver
lung
meninges
oesophagus
ovary
pancreas
Cancer Types
COSMIC Cancer Co-mutation Intra-Class Vs. Inter-Class Contact Frequencies on hESC CellLine
Spatial Contact Frequency
p=4.27e-02
p=5.31e-02
p=3.21e-01
p=2.29e-13
p=5.23e-01
p=3.35e-01
p=3.58e-07
p=9.10e-19
p=1.87e-01
p=2.87e-04
Intra-class
Inter-class
parathyroid
prostate
salivary_gland
skin
small_intestine
soft_tissue
stomach
thyroid
upper_aerodigestive_tract
urinary_tract
Cancer Types

c

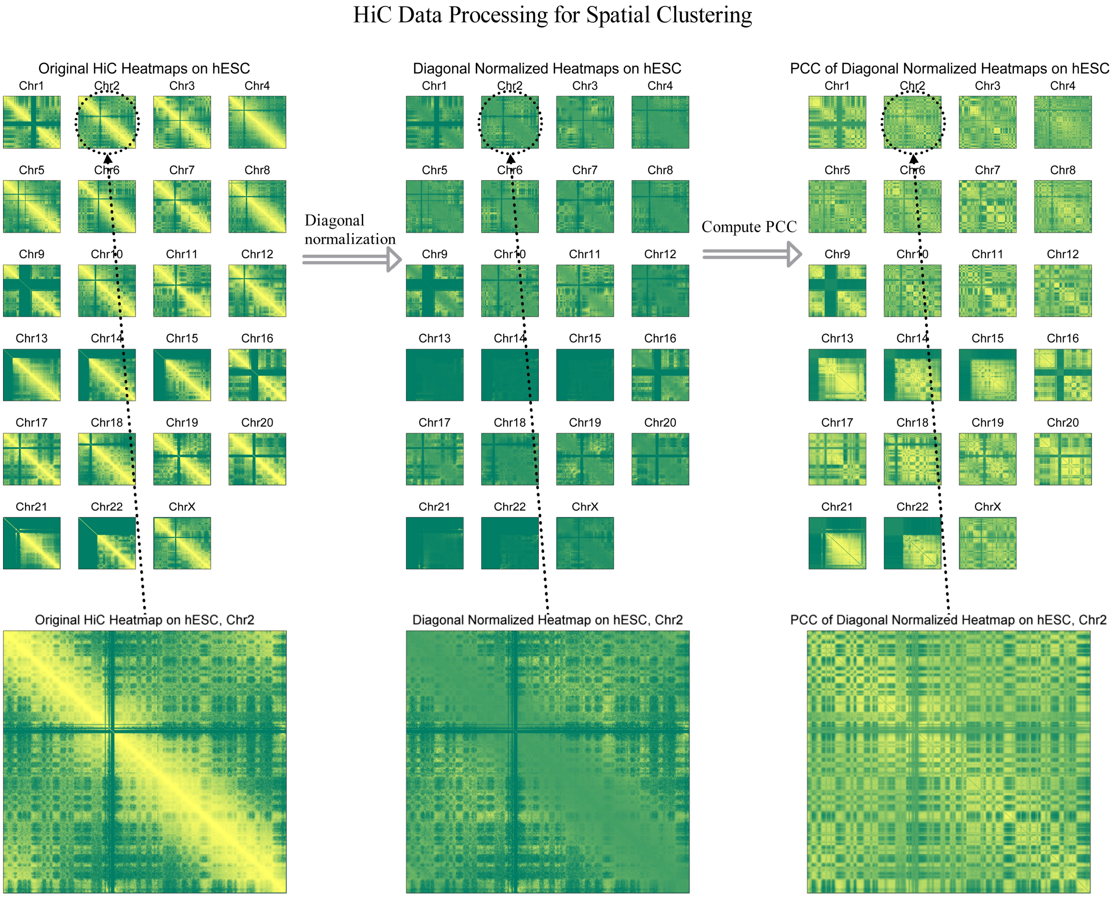

Supplementary Figure 2. Spatial proximities co-mutated gene clusters & Hi-C data process. The comparison of intra-class contact frequency distribution and the inter-class contact frequency distribution. **(a)**. Comparison of intra- and inter-class contact frequency distributions in COSMIC cancers based on IMR90 Hi-C data. **(b)**. Comparison of intra-class and inter-class contact frequency distributions in COSMIC cancers based on hESC Hi-C data. **(c)**. Illustration of the Hi-C data processing procedure. Top left: The original Hi-C heatmaps of each chromosome in the hESC cell line. Top middle: The diagonal-normalised Hi-C heatmaps of each chromosome. Top right: The Pearson correlation coefficient matrices based on the diagonal-normalised Hi-C heatmaps. Bottom: The zoomed-in matrices on chromosome 2.

a

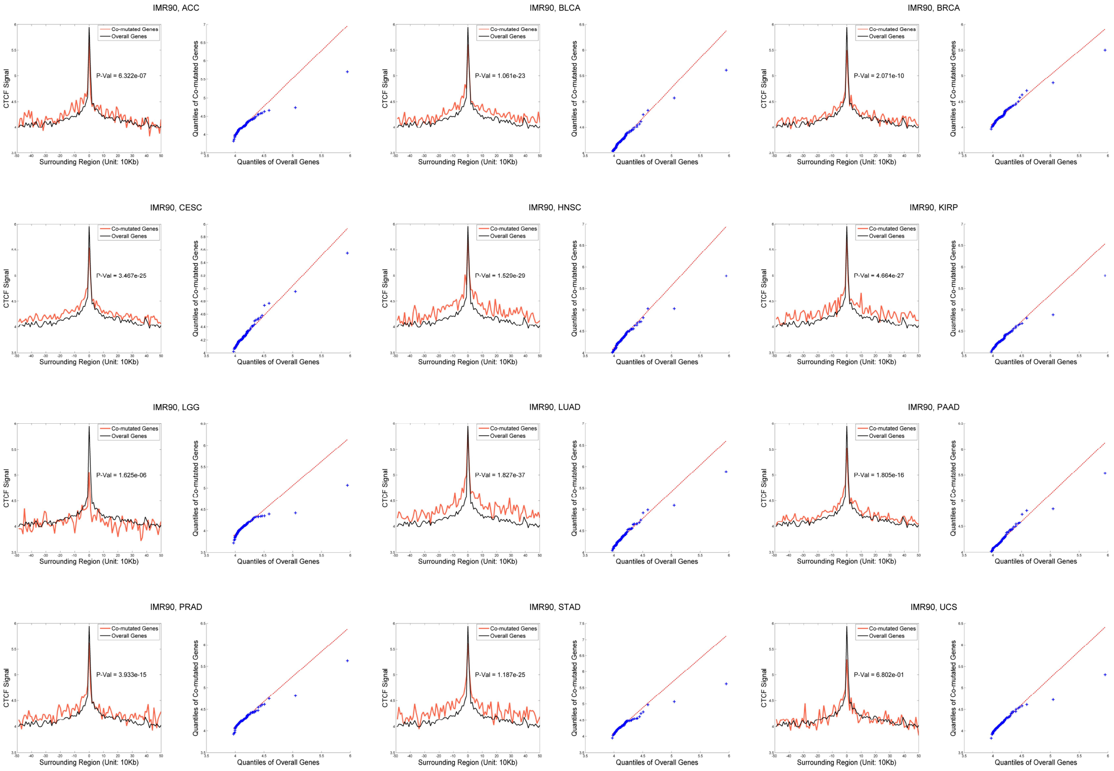

b

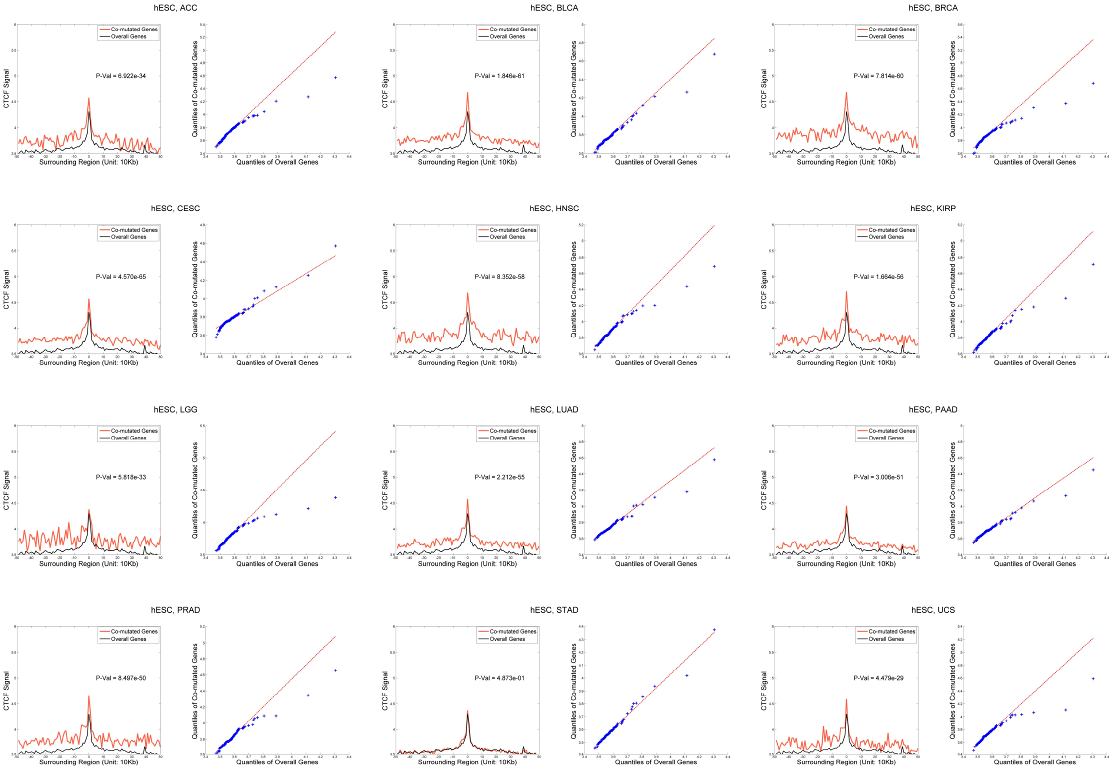

Supplementary Figure 3. CTCF-binding sites near SCH genes. CTCF-binding sites near the transcription start sites (TSS) of SCH genes of 12 TCGA cancer types (red) were statistically compared with that of all genes as background (black) on IMR90 (**a, left**) and

hESC Hi-C data (**b, left**), through employing CTCF ChIP-seq data, where the corresponding quantile-quantile plots of the distributions of their CTCF--binding signals are demonstrated in right. The *T*-test *P*-values are shown on the plots.

a

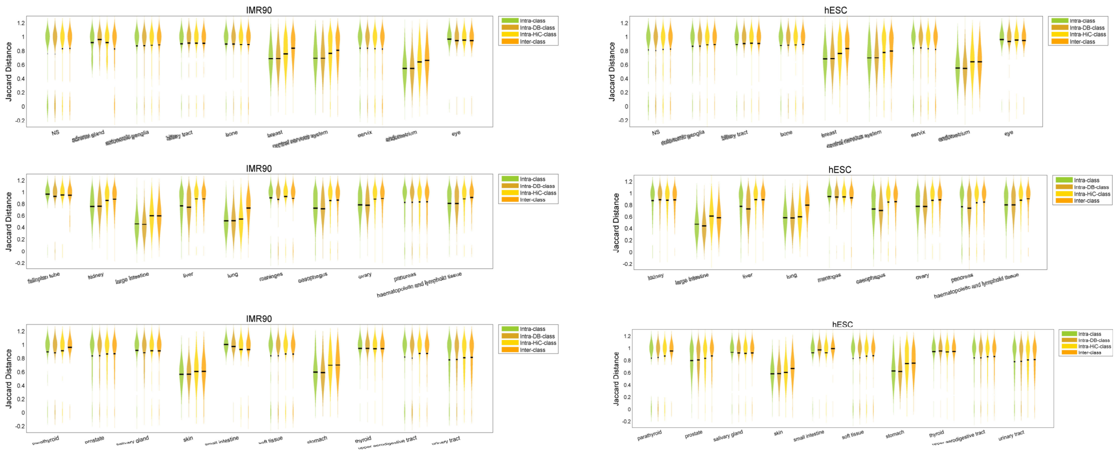
IMR90
hESC
Jaccard Distance
Intra-class
Intra-DB-class
Intra-HiC-class
Inter-class

b

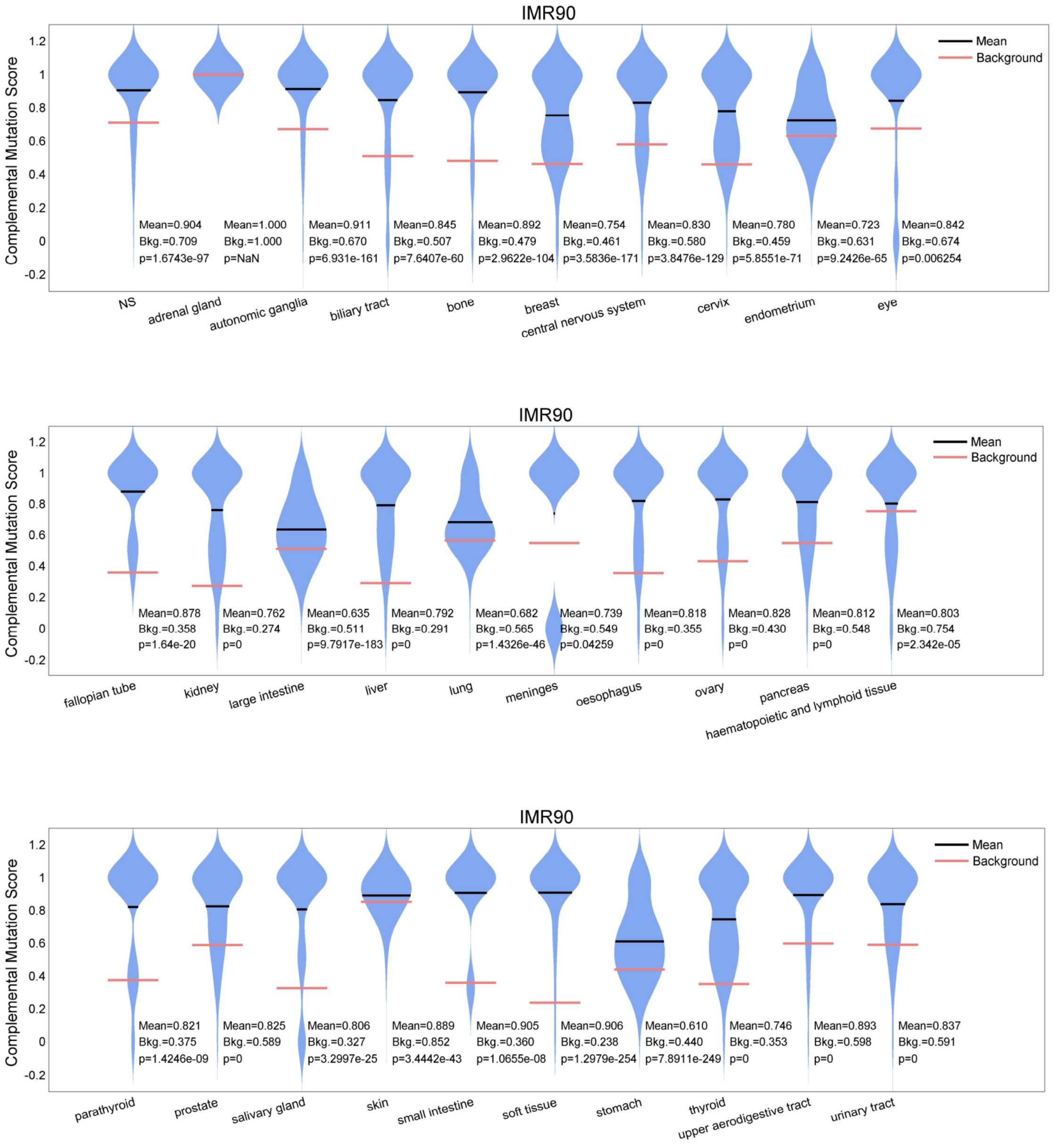
IMR90
Complemental Mutation Score
Mean
Background
Mean=0.904 Bkg.=0.709 p=1.6743e-97
Mean=1.000 Bkg.=1.000 p=NaN
Mean=0.911 Bkg.=0.670 p=6.931e-161
Mean=0.845 Bkg.=0.507 p=7.6407e-60
Mean=0.892 Bkg.=0.479 p=2.9622e-104
Mean=0.754 Bkg.=0.461 p=3.5836e-171
Mean=0.830 Bkg.=0.580 p=3.8476e-129
Mean=0.780 Bkg.=0.459 p=5.8551e-71
Mean=0.723 Bkg.=0.631 p=9.2426e-65
Mean=0.842 Bkg.=0.674 p=0.006254
NS
adrenal gland
autonomic ganglia
biliary tract
bone
breast
central nervous system
cervix
endometrium
eye
IMR90
Complemental Mutation Score
Mean
Background
Mean=0.878 Bkg.=0.358 p=1.64e-20
Mean=0.762 Bkg.=0.274 p=0
Mean=0.635 Bkg.=0.511 p=9.7917e-183
Mean=0.792 Bkg.=0.291 p=0
Mean=0.682 Bkg.=0.565 p=1.4326e-46
Mean=0.739 Bkg.=0.549 p=0.04259
Mean=0.818 Bkg.=0.355 p=0
Mean=0.828 Bkg.=0.430 p=0
Mean=0.812 Bkg.=0.548 p=0
Mean=0.803 Bkg.=0.754 p=2.342e-05
fallopian tube
kidney
large intestine
liver
lung
meninges
oesophagus
ovary
pancreas
haematopoietic and lymphoid tissue
IMR90
Complemental Mutation Score
Mean
Background
Mean=0.821 Bkg.=0.375 p=1.4246e-09
Mean=0.825 Bkg.=0.589 p=0
Mean=0.806 Bkg.=0.327 p=3.2997e-25
Mean=0.889 Bkg.=0.852 p=3.4442e-43
Mean=0.905 Bkg.=0.360 p=1.0655e-08
Mean=0.906 Bkg.=0.238 p=1.2979e-254
Mean=0.610 Bkg.=0.440 p=7.8911e-249
Mean=0.746 Bkg.=0.353 p=0
Mean=0.893 Bkg.=0.598 p=0
Mean=0.837 Bkg.=0.591 p=0
parathyroid
prostate
salivary gland
skin
small intestine
soft tissue
stomach
thyroid
upper aerodigestive tract
urinary tract

c

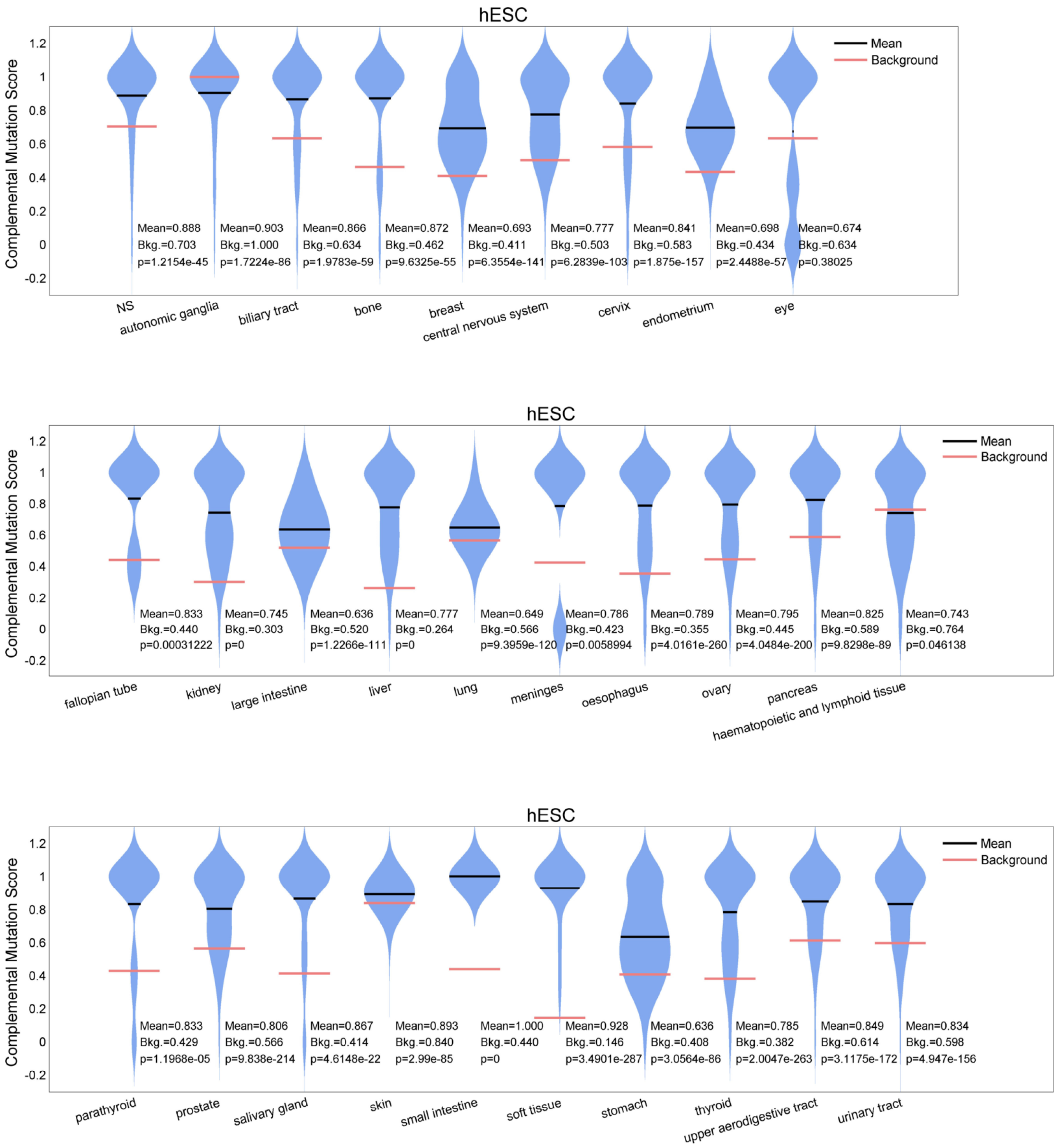

Supplementary Figure 4. Analyses on mutational signatures in co-mutations within SCHs. **(a)**. Jaccard distance distributions of mutational signature profiles comparing intra-class, intra-DB-class, intra-HiC-class and inter-class mutations in COSMIC datasets. **(b)**. Distribution of complementary mutation scores of hotspots in 2 COSMIC cancer cell lines based on IMR90 Hi-C data and compared with the background complementary mutation score. **(c)**. Distribution of complementary mutation scores of hotspots in 2 COSMIC cancer cell lines based on hESC Hi-C data and compared with the background complementary mutation score.

a

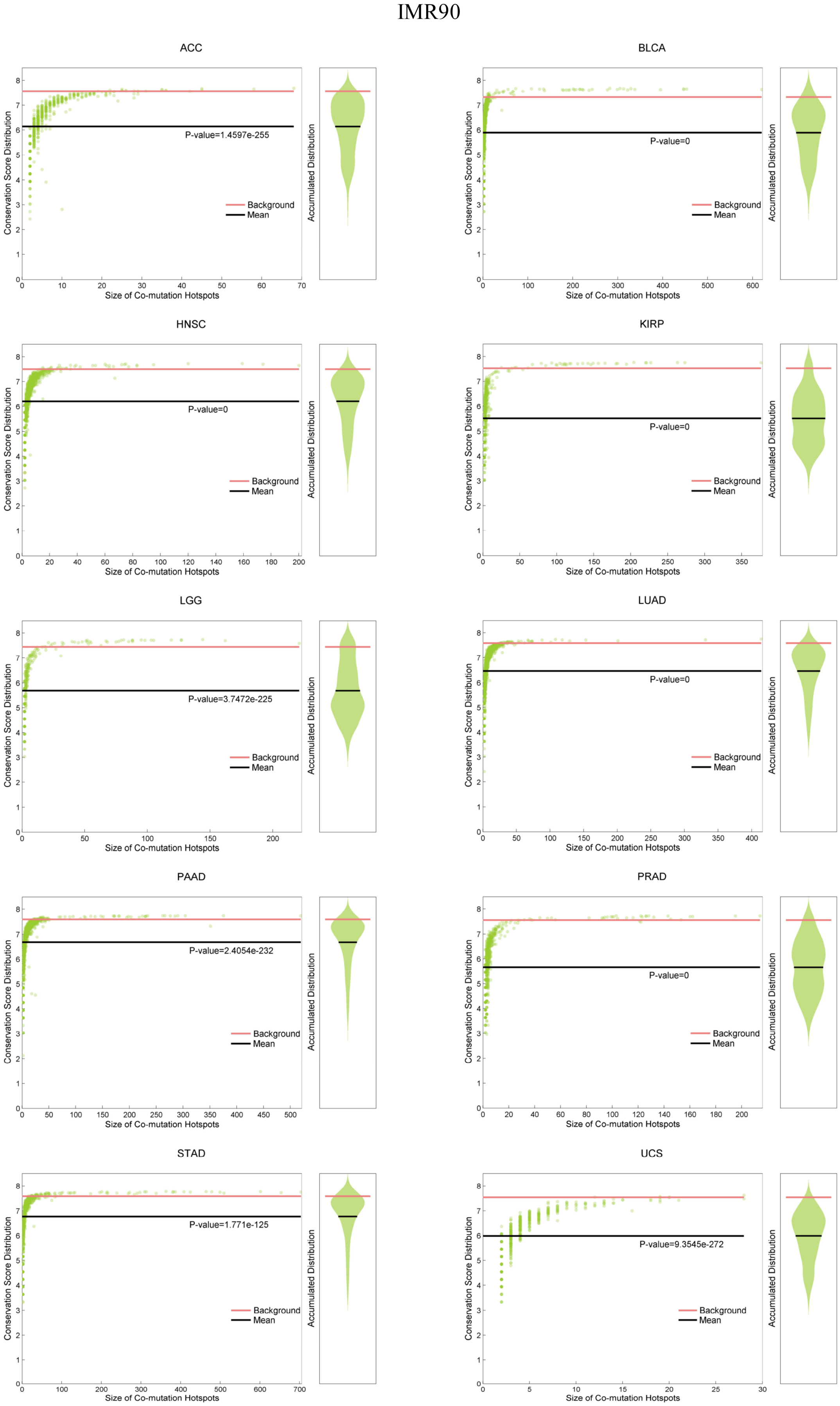
IMR90
ACC
P-value=1.4597e-255
BLCA
P-value=0
HNSC
P-value=0
KIRP
P-value=0
LGG
P-value=3.7472e-225
LUAD
P-value=0
PAAD
P-value=2.4054e-232
PRAD
P-value=0
STAD
P-value=1.771e-125
UCS
P-value=9.3545e-272
Background
Mean
Conservation Score Distribution
Size of Co-mutation Hotspots
Accumulated Distribution

b

hESC

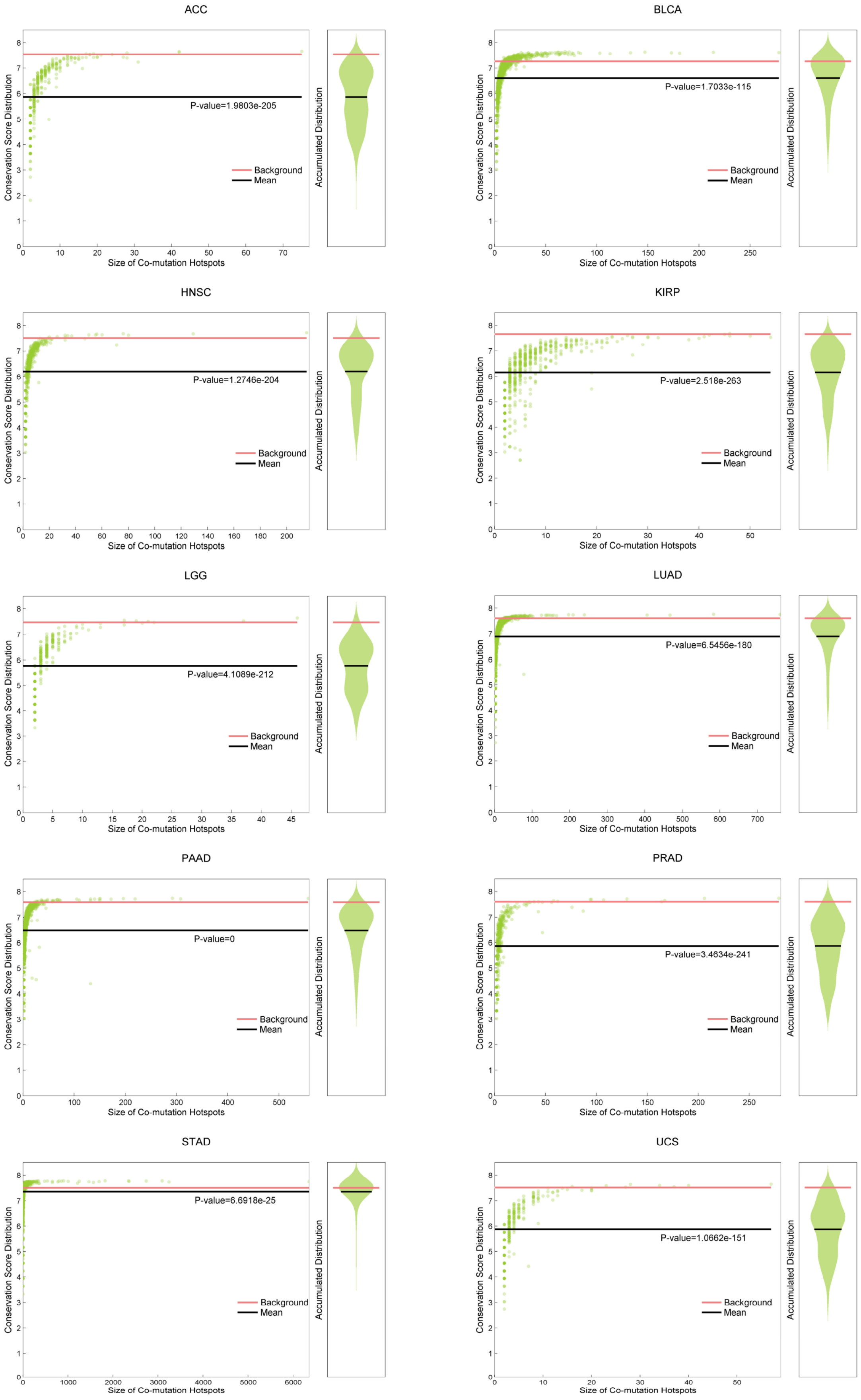
ACC
P-value=1.9803e-205
BLCA
P-value=1.7033e-115
HNSC
P-value=1.2746e-204
KIRP
P-value=2.518e-263
LGG
P-value=4.1089e-212
LUAD
P-value=6.5456e-180
PAAD
P-value=0
PRAD
P-value=3.4634e-241
STAD
P-value=6.6918e-25
UCS
P-value=1.0662e-151
Background
Mean
Conservation Score Distribution
Size of Co-mutation Hotspots
Accumulated Distribution

c

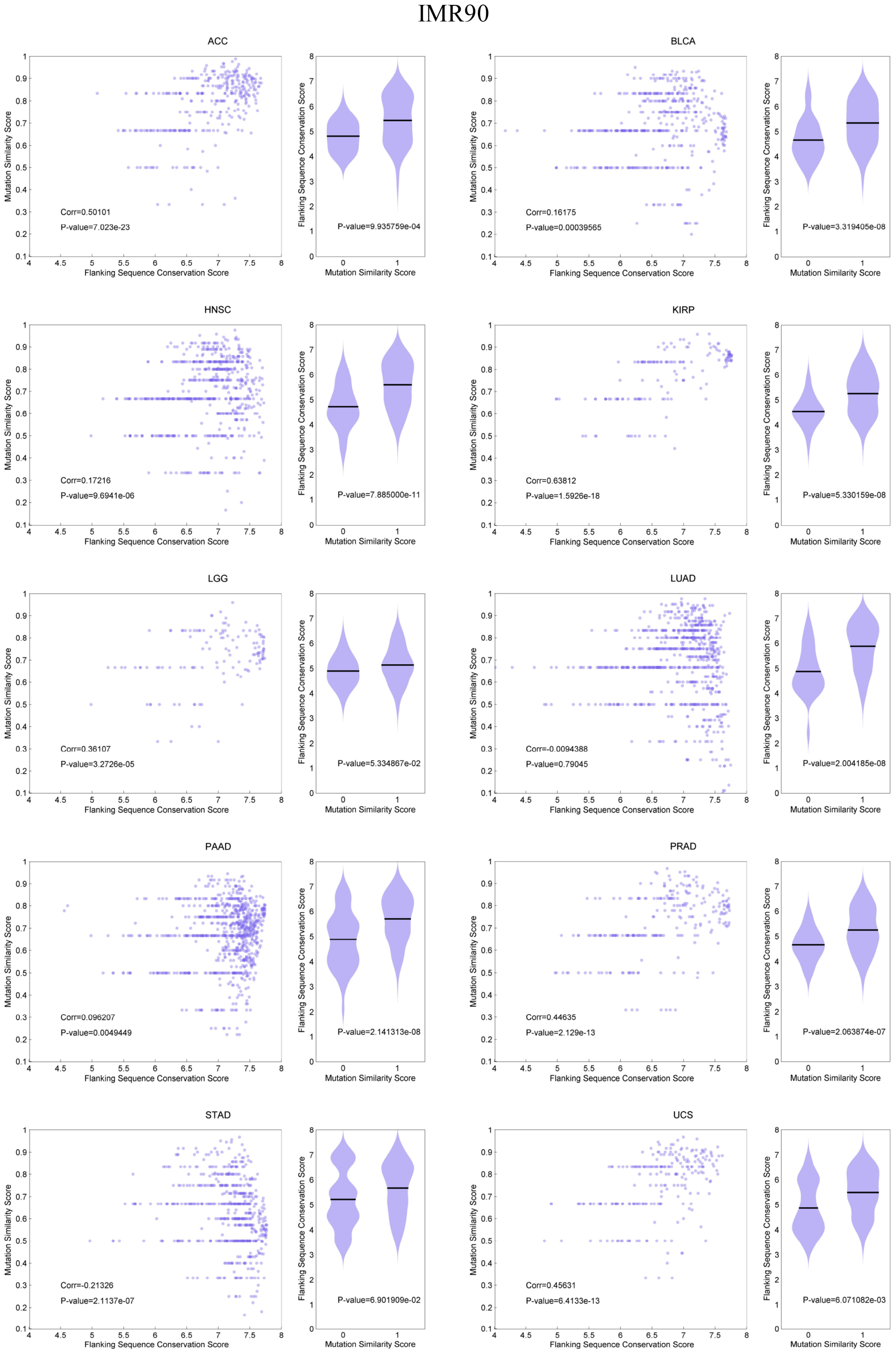

IMR90
ACC
Corr=0.50101
P-value=7.023e-23
P-value=9.935759e-04
BLCA
Corr=0.16175
P-value=0.00039565
P-value=3.319405e-08
HNSC
Corr=0.17216
P-value=9.6941e-06
P-value=7.885000e-11
KIRP
Corr=0.63812
P-value=1.5926e-18
P-value=5.330159e-08
LGG
Corr=0.36107
P-value=3.2726e-05
P-value=5.334867e-02
LUAD
Corr=-0.0094388
P-value=0.79045
P-value=2.004185e-08
PAAD
Corr=0.096207
P-value=0.0049449
P-value=2.141313e-08
PRAD
Corr=0.44635
P-value=2.129e-13
P-value=2.063874e-07
STAD
Corr=-0.21326
P-value=2.1137e-07
P-value=6.901909e-02
UCS
Corr=0.45631
P-value=6.4133e-13
P-value=6.071082e-03
Mutation Similarity Score
Flanking Sequence Conservation Score

d

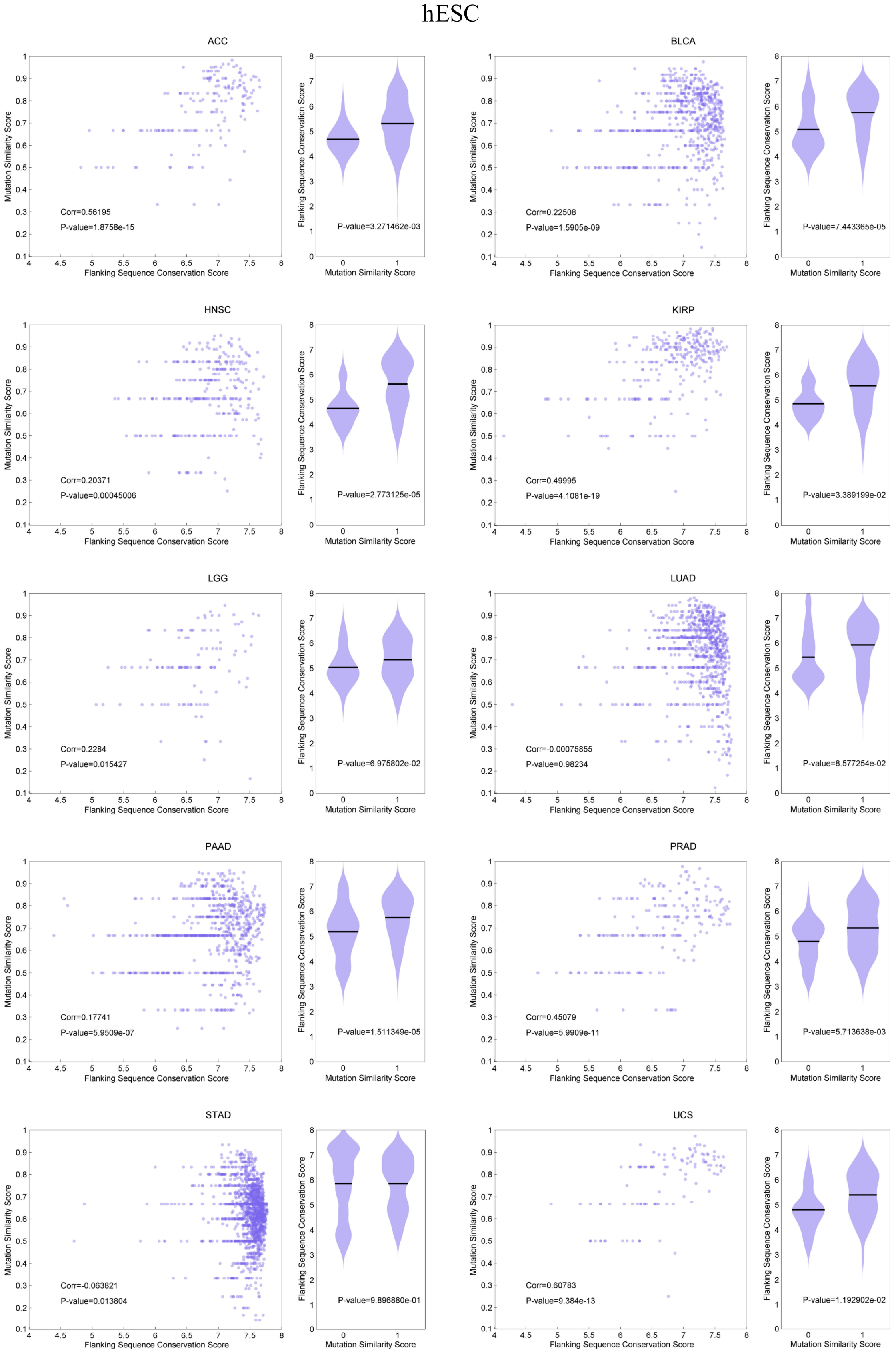

Supplementary Figure 5. Analyses on the flanking sequences neighboring co-mutation points within SCHs of 12 TCGA cancer types. Flanking sequence

conservation test on TCGA cancer datasets with IMR90 (**a**) and hESC (**b**) Hi-C data. The left panels show the relationship between the co-mutation hotspot size (x-axis) and the mean flanking sequence conservation score (y-axis). The corresponding violin plots of the accumulated conservation scores marginalized over SCH sizes was shown in the right panels. Correlation tests between mutational signature similarities and the flanking sequence conservation within hotspots of TCGA cancer types on IMR90 (**c, left**) and hESC (**d, left**) Hi-C data. Comparisons of the flanking sequence conservation score with hotspots of mutation similarity score were shown in the right panels. 0 means identical and 1 means distinct.

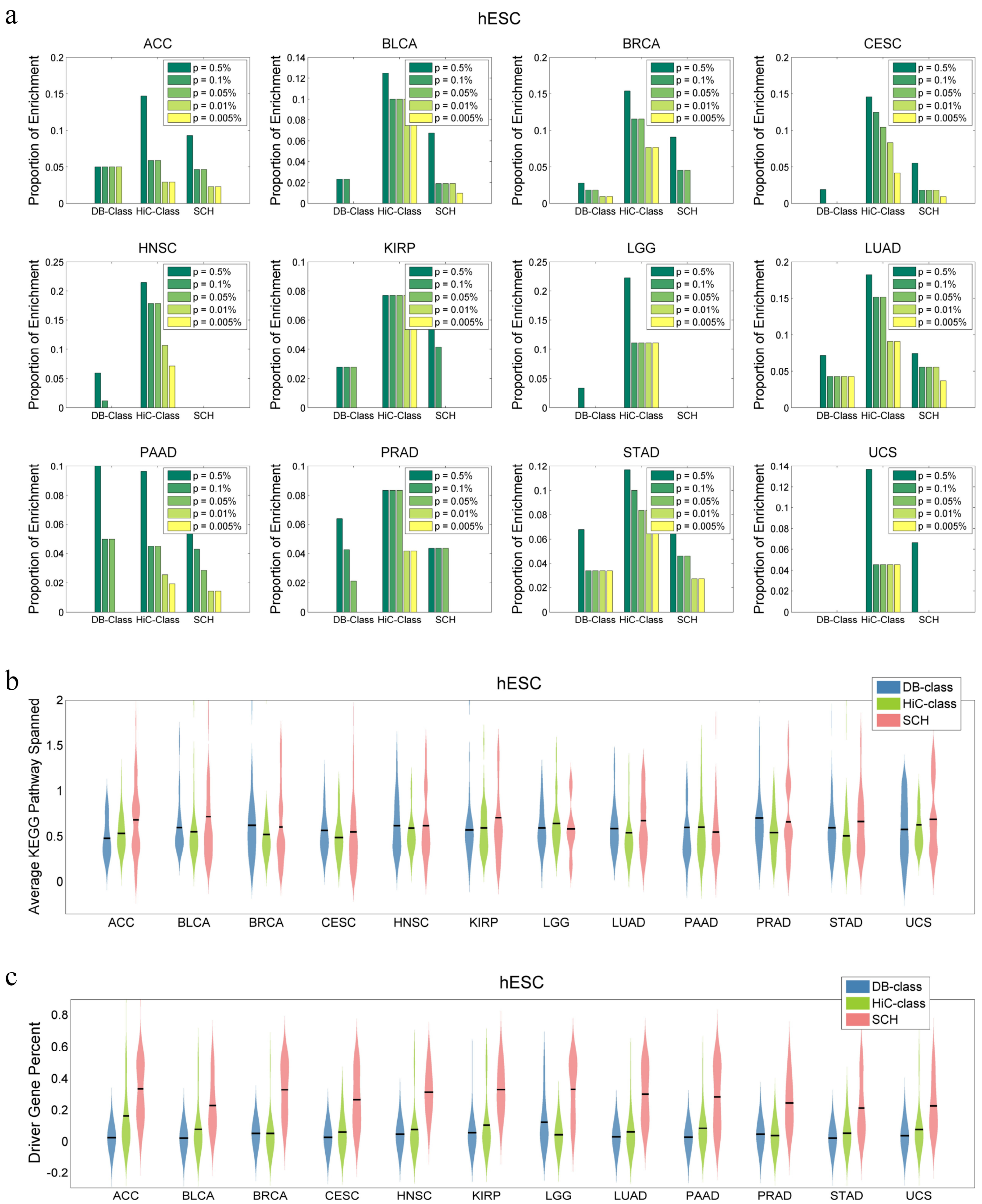

Supplementary Figure 6. Signaling pathway enrichment and cancer driver genes within SCHs based on hESC Hi-C data. (a) The proportions of KEGG signalling pathway enrichment among different gene clusters from 12 TCGA database (DB) cancer types, hESC Hi-C data and SCHs (overlap) were statistically compared using different significance cut-offs. The average numbers of spanned KEGG pathways **(b)** and the percentages of cancer driver genes **(c)** among the above three resources were also statistically compared in 12 TCGA cancer types.
